# Drp1-Mediated Mitochondrial Metabolic Dysfunction Inhibits the Tumor Growth of Pituitary Adenomas

**DOI:** 10.1155/2022/5652586

**Published:** 2022-03-23

**Authors:** Kexia Fan, Xiao Ding, Zhenle Zang, Yin Zhang, Xiaoshuang Tang, Xiangdong Pei, Qingbo Chen, Huachun Yin, Xin Zheng, Yong Chen, Song Li, Hui Yang

**Affiliations:** ^1^Multidisciplinary Center for Pituitary Adenomas of Chongqing, Department of Neurosurgery, Xinqiao Hospital, Army Medical University, Chongqing, China; ^2^Chongqing Institute for Brain and Intelligence, Guangyang Bay Laboratory, Chongqing, China

## Abstract

Metabolic changes have been suggested to be a hallmark of tumors and are closely associated with tumorigenesis. In a previous study, we demonstrated the role of lactate dehydrogenase in regulating abnormal glucose metabolism in pituitary adenomas (PA). As the key organelle of oxidative phosphorylation (OXPHOS), mitochondria play a vital role in the energy supply for tumor cells. However, few attempts have been made to elucidate mitochondrial metabolic homeostasis in PA. Dynamin-related protein 1 (Drp1) is a member of the dynamin superfamily of GTPases, which mediates mitochondrial fission. This study is aimed at investigating whether Drp1 affects the progression of PA through abnormal mitochondrial metabolism. We analyzed the expression of dynamin-related protein 1 (Drp1) in 20 surgical PA samples. The effects of Drp1 on PA growth were assessed in vitro and in xenograft models. We found an upregulation of Drp1 in PA samples with a low proliferation index. Knockdown or inhibition of Drp1 enhanced the proliferation of PA cell lines in vitro, while overexpression of Drp1 could reversed such effects. Mechanistically, overexpressed Drp1 damaged mitochondria by overproduction of reactive oxygen species (ROS), which induced mitochondrial OXPHOS inhibition and decline of ATP production. The energy deficiency inhibited proliferation of PA cells. In addition, overexpressed Drp1 promoted cytochrome c release from damaged mitochondria into the cytoplasm and then activated the downstream caspase apoptotic cascade reaction, which induced apoptosis of PA cells. Moreover, the decreased ATP production induced by Drp1 overexpressing activated the AMPK cellular energy stress sensor and enhanced autophagy through the AMPK-ULK1 pathway, which might play a protective role in PA growth. Furthermore, overexpression of Drp1 repressed PA growth in vivo. Our data indicates that Drp1-mediated mitochondrial metabolic dysfunction inhibits PA growth by affecting cell proliferation, apoptosis, and autophagy. Selectively targeting mitochondrial metabolic homeostasis stands out as a promising antineoplastic strategy for PA therapy.

## 1. Introduction

Pituitary adenomas (PA) are common intracranial tumors with an incidence of approximately 10-15% in primary intracranial tumors [1]. The 2017 WHO classification of PA has emphasized the pathologic evaluation of tumor proliferative potential for the identification of clinically aggressive adenomas [2]. As proliferative activity increases, a subset of PA cases gradually shows malignant features such as rapid local growth and tumor extension, which make total resection difficult and result in early recurrence [3]. Accordingly, it is crucial for developing novel strategies to attenuate the proliferative activity of PA to improve patient prognosis.

Metabolic changes are closely associated with tumorigenesis [4] and are one of the hallmarks of tumors [5]. Reprogramming of cellular metabolism is an important characteristic of tumors [6]. Aberrant hormone secretion is conspicuous in most PA, which causes significant metabolic reprogramming in PA cells. In turn, these metabolic alterations may influence the progression of PA [7]. In a previous study, we demonstrated the role of lactate dehydrogenase in regulating abnormal glucose metabolism in PA [8].

As the vital organelle for glucose metabolism, mitochondria play a pivotal role in tumor cellular energy supply through oxidative phosphorylation (OXPHOS) [9–11]. In addition, mitochondria not only provide building blocks for tumor anabolism, control redox, and calcium homeostasis but also participate in the regulation of oncogenic transcription and tumor cell death [12]. Therefore, mitochondria constitute promising targets for the development of novel antitumor therapies in studies of various tumors [13, 14]. Currently, the changes of mitochondrial metabolism during PA progression are not well understood.

Dynamin-related protein 1 (Drp1) is a member of the dynamin superfamily of GTPases. Drp1 mediates fission of the mitochondria [15–17], which permits renewal and redistribution of metabolites inside the cells [18, 19]. Drp1 has been shown to affect tumor progression in many tumor types [20–24]. However, whether Drp1 can affect the PA progression by directly regulating mitochondrial metabolism remains unknown.

In the current study, we evaluated the expression of Drp1 in human PA samples and analyzed the effects of Drp1 on cell proliferation and apoptosis by using PA cell lines. Furthermore, we explored the potential mechanism by which Drp1 affects PA growth, specifically focusing on mitochondrial metabolism. Moreover, we verified our findings by using PA xenograft model in vivo. Our study provides new insight into the mechanism underlying PA progression and antineoplastic strategy for targeting mitochondrial metabolism in PA treatment.

## 2. Materials and Methods

### 2.1. Patients

Twenty human PA samples were collected from patients who underwent transsphenoidal surgery at the multidisciplinary center for PA in Chongqing, Xinqiao Hospital. Individual tumors were diagnosed based on clinical, endocrine, and pathological evaluations. According to the prognostic clinicopathological classification of PA described by Trouillas et al. [25], high proliferation index PA (HI-PA, *n* = 10) tumors were identified by a Ki-67 index ≥ 3%, accompanied by P53 positivity; low proliferation index PA (LI-PA, *n* = 10) tumors were identified by a Ki-67 index < 3%, accompanied by P53 negativity. Clinical data regarding the gender, age, and PA hormone type of all patients are summarized in Table S1.

### 2.2. Reagents

The following reagents were obtained: human pituitary gland RNA standard (Clontech, 636157) and puromycin (Beyotime, ST551). The following reagents were purchased from MedChemExpress: Mdivi-1 (HY-15886), Mito-TEMPO (HY-112879), cyclosporin A (CsA; HY-B0579), Compound C (CC; HY-13418A), and 3-methyladenine (3-MA; HY-19312). Primary antibodies for western blotting were used: Drp1 (Abcam, ab184247), caspase-9 (Abcam, ab184786), Bcl-2 (Proteintech, 26593-1-AP), p-IRE1 (Bioss, bs-16698R), XBP1s (Proteintech, 24868-1-AP), p-PERK (Bioss, bs-3330R), and ATF6 (Abcam, ab37149). The following primary antibodies were purchased from Cell Signaling Technology: cytochrome c (11940), caspase-3 (14220), PARP (9532), Bax (2772), Cyclin D1 (2978), P-AMPK (2535), AMPK (5831), P-ULK1 (37762), ULK1 (8054), LC3 (43566), p62 (5114), p-eIF2*α* (3398), *β*-actin (4970), and VDAC (4661). The Goat anti-rabbit secondary antibody for western blotting was obtained from Zsbio (ZB-2301).

### 2.3. Cell Culture

Rat GH3 (ATCC, CCL-82.1), rat MMQ (ATCC, CRL-10609), and mouse AtT-20 (ATCC, CCL-89) PA cell lines were cultured in Ham's F-12 K medium (Gibco, 21127-022) containing 2.5% fetal bovine serum (Gibco, 10100-147) and 15% horse serum (Gibco, 16050-122) in a humidified atmosphere of 5% CO_2_ at 37°C.

### 2.4. Reverse Transcription and qPCR

Total RNA was extracted using TRIzol Reagent (TaKaRa, 9108), and 1 *μ*g of total RNA was reverse transcribed to cDNA using a PrimeScript RT Reagent Kit (TaKaRa, PR047A). TB Green Premix Ex Taq II (TaKaRa, RR820A) and a CFX96 Real-time System (Bio-Rad Laboratories) were used to carry out qPCR. The relative expression levels were calculated using the 2 −  ^△△ct^ method. The primer sequences used for qPCR are listed in Table S2.

### 2.5. Western Blotting Analysis

Total proteins, cytosolic proteins, and mitochondrial proteins were analyzed using western blotting. Cell extracts containing 50 *μ*g of protein were subjected to SDS-PAGE (Boster, AR0138) and transferred to PVDF membranes (Millipore, ISEQ00010). After blocking, membranes were incubated overnight with primary antibodies (1 : 1000). Membranes were probed with secondary antibody (1 : 2500). The signals from the membrane were detected by enhanced chemiluminescence.

### 2.6. Immunohistochemistry

Tumor tissue was fixed in 4% paraformaldehyde for 24 h and embedded in paraffin. Embedded tumor tissues were sectioned at 5 *μ*m for subsequent immunohistochemical (IHC) staining. IHC staining was performed according to standard protocols. Drp1 primary antibody (1 : 500, Abcam, ab184247) was used. Microscopy (MSHOT, MF53) was used to obtain images of the different sections.

### 2.7. Lentiviral and shRNA Transfection

Empty and Drp1-overexpressing lentiviral vectors (GeneChem) were transfected into GH3 cells at a multiplicity of infection of 100 according to the manufacturer's instructions. Stable colonies were selected by adding puromycin (2 *μ*g/ml) to the transfectants for 72 h and separating cells with fluorescent labeling using a flow cytometer (Beckman Coulter, Gallios). Transfection efficiency was determined by RT-qPCR and western blotting.

GH3 cells were transfected with negative control (NC) or Drp1 gene knockdown shRNA vectors (Genechem) using Lipofectamine 2000 (Invitrogen Life Technologies, 11668-027) according to the manufacturer's instructions. Fluorescently labeled cells were isolated by flow cytometry. Transfection efficiency was determined by RT-qPCR. The same treatment was carried out with cytochrome c gene (Cycs).

The short interfering RNAs (siRNAs) targeting AMPK were purchased from Santa Cruz Biotechnology. GH3 cells were transfected with siRNAs containing AMPK*α*1 siRNA (sc-270142) and AMPK*α*2 siRNA (sc-155985) or transfected with control (Ctrl) siRNA (sc-37007) for 24 h using siRNA transfection reagent (sc-29528) according to the manufacturer's instructions. Transfection efficiency was determined by RT-qPCR.

The target sequences of shRNAs and siRNAs are listed in Table S3.

### 2.8. Cell Proliferation Assay

A WST-8 Cell Counting Kit-8 (CCK-8; Dojindo, CK04) was used to measure cell proliferation. Cells were seeded at 2 × 10^4^ cells/well into 96-well plates with 100 *μ*l of culture medium. Ten microliters of CCK-8 reagent was added to the wells at specific time points and was incubated for 2 h at 37°C. The reaction product was quantified according to the manufacturer's instructions.

### 2.9. Cell Apoptosis Measurement

For cell apoptosis analysis, cells were collected at the indicated time points for the relevant treatments. Apoptosis was assessed using a FITC-Annexin V Apoptosis Detection Kit (BD Biosciences Pharmingen, 556547) based on the manufacturer's instructions, and cells were detected by flow cytometry.

### 2.10. Transmission Electron Microscopy (TEM)

Thirty-six GH3 cell samples for TEM were obtained by using a conventional sample preparation protocol. Briefly, cells were harvested and fixed with 2.5% glutaraldehyde and then postfixed with 1% osmium tetroxide. The pellets were dehydrated in graded concentrations of ethanol, infiltrated, and embedded in araldite. Samples were then cut into ultrathin sections (60 nm) and stained with aqueous uranyl acetate and lead citrate. Images were acquired using a JEM-1200 electron microscope (JEOL).

### 2.11. Mitochondrial Membrane Potential Assay

TMRE accumulates in hyperpolarized mitochondria, and the fluorescence intensity correlates with mitochondrial membrane potential (MMP) [26]. MMP assay kit with TMRE was purchased from Beyotime Biotechnology (C2001S). GH3 cells were incubated with 1X TMRE for 30 min at 37°C in the dark. Then, the cells were washed twice with PBS and incubated in culture medium. Cells were visualized within 1 h by using a Leica fluorescence microscope. Fluorescence intensity was analyzed by ImageJ.

### 2.12. Mitochondrial Respiration and Glycolysis

Mitochondrial respiration capacity was detected by the oxygen consumption rate (OCR), which was measured by using the Seahorse XF96 extracellular analyzer (Agilent) according to the manufacturer's protocol. Briefly, 2 × 10^4^ cells were seeded in a 96-well Seahorse XF cell culture microplate (Agilent, 101085) for 24 h in culture medium. Cells were then incubated in XF assay media (XF base medium, Agilent, 102353; 100 mM pyruvate, Sigma, S8636; 200 mM glutamine, Gibco, 25030; 2.5 M glucose, Sigma, G8769) for 45 min at 37°C in a non–CO_2_ incubator. Cells were then transferred to the Seahorse analyzer at 37°C and sequentially treated with 1 *μ*M oligomycin, 0.5 *μ*M FCCP, and a 0.5 mM mixture of rotenone and antimycin A, which were contained in the Seahorse XF Cell Mito Stress Test Kit (Agilent, 103015). The OCR was automatically measured after the addition of each compound as the average of two readings from three wells. At the end of each experiment, the amount of protein in each well was quantified, and the OCR values were corrected to avoid differences caused by variations in the number of cells.

Glycolysis capacity was detected by the extracellular acidification rate (ECAR), which was measured by using the Seahorse XF96 extracellular analyzer according to the manufacturer's protocol. Most of the steps were the same as those used for the experiment detecting OCR, but the XF assay media was made up of XF base medium (Agilent, 102353) and 200 mM glutamine (Gibco, 25030). In the Seahorse analyzer, cells were sequentially treated with 10 mM glucose, 1 *μ*M oligomycin, and 50 mM 2-DG, which were contained in the Seahorse XF Glycolysis Stress Test Kit (Agilent, 103020). Schematic diagrams of the parameters for OCR and ECAR are presented in Figure S1.

### 2.13. ATP Measurement

Intracellular ATP was measured using an ATP Assay kit (Beyotime, S0026) according to the manufacturer's instructions. The luminescence produced was measured with Thermo Scientific Luminoskan Ascent, and the concentration of ATP was calculated using an ATP standard curve.

### 2.14. Mitochondria Isolation

Mitochondria were isolated from GH3 cells and xenograft tumors. The collected cell pellets were washed twice with cold PBS, and mitochondrial isolation was carried out using a cell mitochondria isolation kit (Beyotime, C3601). Briefly, cells were resuspended in mitochondrial isolation buffer (provided in the kit) and homogenized with 15 strokes of a homogenizer. The obtained suspensions were centrifuged at 600 × g for 10 min, and the supernatant was collected and centrifuged at 11,000 × g for 10 min to pellet the mitochondria. The precipitated mitochondria were collected and extracted as mitochondrial fractions. The supernatant was centrifuged at 12,000 × g for 10 min and collected as cytosolic fractions. Utilizing a tissue mitochondria isolation kit (Beyotime, C3606), the intact mitochondria of excised tumors were isolated from xenograft model mice. Briefly, after homogenization of tumor tissue in ice-cold buffer provided in the kit, the homogenate was centrifuged at 600 × g for 5 min. The rest steps were the same as those used for the isolation of cell mitochondria. These steps were performed at 4°C, and then, mitochondrial and cytosolic fractions were stored at −80°C until use.

### 2.15. Analysis of Reactive Oxygen Species (ROS)

Intracellular ROS content was measured using the Reactive Oxygen Species Assay Kit (Beyotime, S0033) according to the manufacturer's protocol. Briefly, after incubation with 10 *μ*M DCFH-DA for 20 min at 37°C in darkness, each sample was assessed for fluorescence intensity by flow cytometry. The same treatment was carried out with isolated mitochondria to detect mitochondrial ROS. The data of intracellular and mitochondrial ROS were used to calculate cytosolic ROS.

### 2.16. Immunofluorescence

Immunofluorescence was performed according to standard protocols. Cells fixed in 4% paraformaldehyde were incubated at 4°C overnight with a primary antibody against Drp1 (1 : 50; Santa Cruz, sc-271583), TOMM20 (1 : 250; Abcam, ab186735), and cytochrome c (1 : 500; Beyotime, AC908). After washing three times with PBS, cells were stained with Goat anti-mouse secondary antibody (1 : 100; Boster, BA1126) and Goat anti-rabbit secondary antibody (1 : 100; Boster, BA1142). Antifade mounting medium with DAPI (Beyotime, P0131) was used for nuclear staining and fluorescence decay resistance. The fluorescence was excited and imaged by a laser scanning confocal microscopy (SP5, Leica).

### 2.17. In Vivo Experiments

In in vivo xenograft experiments, twenty-four 4-week-old male BALB/cA-nu mice were purchased from Beijing HFK Bioscience Co. Ltd. (China) and housed under SPF conditions. A total of 5 × 10^6^ transfected GH3 cells suspended in 100 *μ*l of solution (50% PBS and 50% Matrigel) were subcutaneously inoculated into the right flank of the mice. Treatment with Mdivi-1 was started 9 days after inoculation of the cells. Then, the tumor-bearing animals were randomly divided into 4 groups (6 mice/group). The Mdivi-1-treated groups (Empty vector + Mdivi-1, Drp1-OE + Mdivi-1) received daily intraperitoneal injection of 50 mg/kg Mdivi-1 for the next 3 weeks until sacrifice [27], while the other two groups (Empty vector, Drp1-OE) received daily intraperitoneal injection of an equal volume of PBS only. The mice were monitored daily for any discomfort. The mice were weighed, and tumor volumes were also measured every three days. Tumor volumes were calculated using the following formula: *V* (mm^3^) = [*AB*^2^]/2, where *A* is the tumor length and *B* is the tumor width. Tumor tissue was removed from tumor-bearing mice following the final treatment. The excised tumors were weighted. Then, the protein was extracted from the excised tumors. All animal procedures were conducted according to protocols approved by the Institutional Animal Care and Ethics Committee.

### 2.18. Statistical Analysis

The data are expressed as the means ± SEMs. A two-tailed Student's *t*-test was applied to determine statistical significance between the two groups. These analyses were performed using SPSS for Windows, version 13.0 (SPSS Inc., USA). ∗*P* < 0.05; ∗∗*P* < 0.01; ∗∗∗*P* < 0.001; #, not significant.

## 3. Results

### 3.1. Drp1 Expression Was Increased in Human LI-PA Samples and Overexpressed Drp1 Affected the Proliferation and Apoptosis of PA Cell Lines

**Figure 1:**
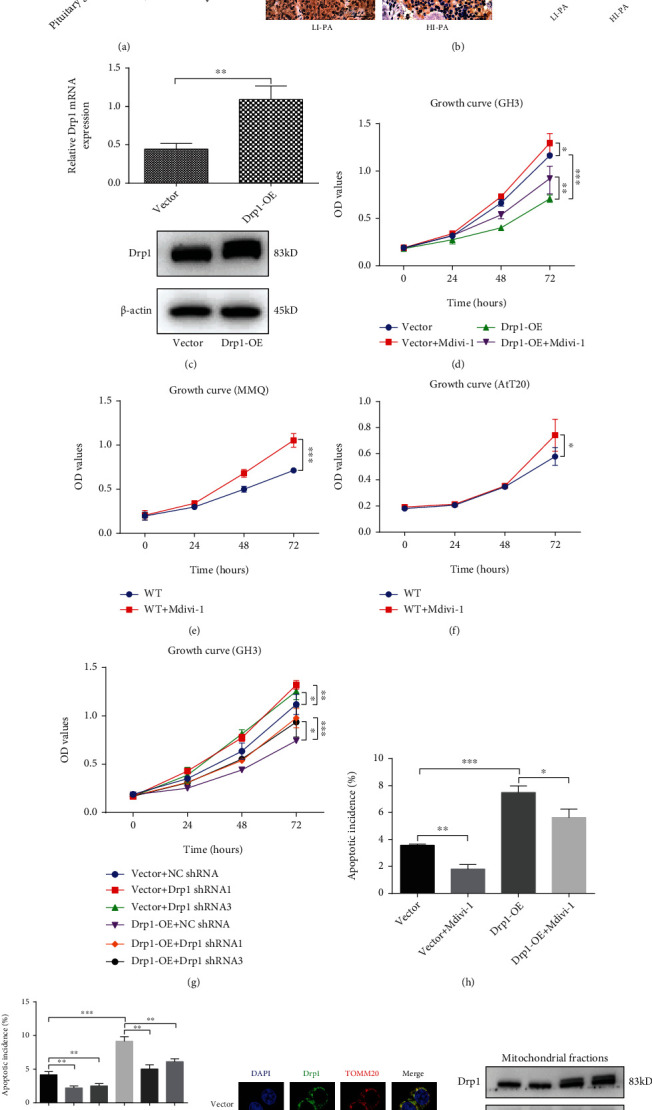
Drp1 expression was increased in human LI-PA samples, and overexpressed Drp1 affected the proliferation and apoptosis of PA cell lines. (a) Expression of Drp1 mRNA was assessed by RT-qPCR in human pituitary gland RNA standard, low proliferation index PA (LI-PA) tissues (*n* = 10), and high proliferation index PA (HI-PA) tissues (*n* = 10). (b) Expression of the Drp1 protein was assessed by IHC staining (left) in LI-PA (*n* = 10) and HI-PA (*n* = 10) sections. Statistical analysis of the IHC staining (right). Scale bar, 50 *μ*m. (c) Drp1 mRNA and protein levels were measured in the control (Vector) group and Drp1-overexpressing (Drp1-OE) group GH3 cells by RT-qPCR and western blotting. (d) Vector and Drp1-OE GH3 cells were incubated with/without 10 *μ*M Mdivi-1 for 0, 24, 48, or 72 h, and cell proliferation was assessed at different time points by a CCK-8 assay (*n* = 6, ± SEM) in each group. (e) Wild-type (WT) MMQ cells were incubated with/without Mdivi-1 (10 *μ*M) for 0, 24, 48, or 72 h. Cell proliferation of the two groups at different time points was assessed by a CCK-8 assay (*n* = 6, ± SEM). (f) The treatment to MMQ cells was carried out with wild-type AtT-20 cells. (g) Vector and Drp1-OE GH3 cells were infected with NC shRNA or Drp1 shRNA (Drp1 shRNA1 or Drp1 shRNA3). Then, cell proliferation was assessed at 0, 24, 48, or 72 h by a CCK-8 assay in each group (*n* = 6, ± SEM). (h) Vector and Drp1-OE GH3 cells were incubated with/without 10 *μ*M Mdivi-1 for 48 h. Cell apoptosis was measured by flow cytometry in each group (*n* = 3, ± SEM). (i) Vector and Drp1-OE GH3 cells were infected with NC shRNA or Drp1 shRNA (Drp1 shRNA1 or Drp1 shRNA3). Cell apoptosis was measured by flow cytometry in each group (*n* = 3, ± SEM). (j) The colocalization of Drp1 and mitochondria in GH3 cells by using immunofluorescence staining. Representative immunostaining of Drp1 (Green) and TOMM20 (Red) was observed by laser confocal microscopy in the vector and Drp1-OE groups. Yellow points represent overlay of Drp1 and mitochondria. Scale bar, 7.5 *μ*m. (k) Protein expression level of Drp1 in isolated mitochondria was detected by western blotting in the vector and Drp1-OE groups. An unpaired *t*-test was used to assess statistical significance. ^∗^*P* < 0.05; ^∗∗^*P* < 0.01; ^∗∗∗^*P* < 0.001.

First, we investigated the expression of Drp1 in 20 surgical PA samples. The RT-qPCR results indicated that the expression of Drp1 mRNA was dramatically upregulated in normal pituitary glands compared with PA samples. Moreover, the Drp1 mRNA level of LI-PA samples was significantly higher than that in HI-PA samples (Figure 1(a)). Further IHC results indicated that LI-PA samples exhibited a higher staining intensity than HI-PA samples (Figure 1(b), left). The statistical results confirmed the upregulated expression of Drp1 protein in LI-PA samples (Figure 1(b), right). These data suggested that the expression level of Drp1 may be associated with the development of PA. Subsequently, cell experiments were performed to verify the proliferative role of Drp1 in PA cell lines, which were assessed by CCK-8 assay. Overexpression of Drp1 (Drp1-OE) in GH3 cell lines by lentiviral vector delivery led to stable upregulation of Drp1 (Figure 1(c); Figure S2(a)). Drp1-OE treatment significantly attenuated the proliferation of GH3 cells. Mdivi-1, a specific inhibitor of Drp1 [28], could reversed the proliferative inhibition by Drp1-OE (Figure 1(d)). Besides, Mdivi-1 also significantly enhanced the proliferation of MMQ and AtT20 cell lines (Figures 1(e) and 1(f)). To further confirm the effect of Mdivi-1, Drp1 shRNAs were used to knock down the expression of Drp1, and the transfection efficiency was confirmed by RT-qPCR (Figure S3(a)). The CCK-8 assay results showed that downregulation of Drp1 expression reversed the repressed effects of Drp1-OE on GH3 cell proliferation (Figure 1(g)). We further assessed the impact on GH3 cell apoptosis. The percentage of apoptotic cells was significantly increased in the Drp1-OE group, which could be reversed by Mdivi-1 (Figure 1(h), Figure S4(a)) and Drp1 shRNAs (Figure 1(i), Figure S4(b)). Considering that Drp1 is the key regulatory protein of mitochondrial division [15, 16], Drp1 recruitment was measured in GH3 cells by immunofluorescence and western blotting, respectively. The results showed that Drp1-OE treatment induced Drp1 recruitment to the mitochondria, as indicated by increased colocalization of Drp1 and TOMM20 (a mitochondrial marker) (Figure 1(j)) and by increased Drp1 protein expression in mitochondrial fraction (Figure 1(k); Figure S2(b)). The endoplasmic reticulum (ER) tubules mark sites of Drp1-mediated mitochondrial division [29, 30]. We measured the key molecules (e.g., p-IRE1, XBP1s, p-PERK, p-eIF2*α*, and ATF6) in classical ER stress-related unfolded protein response (UPR) signalling pathways [31–33]. We found that there were no significant differences in the protein levels of mentioned ER stress-related molecules between the control (vector) and Drp1-OE groups (Figure S5), which indicated that Drp1 overexpression might not affect the ER stress in PA cells. These results indicated that Drp1 could regulate the proliferation and apoptosis by acting on mitochondria in PA cells.

### 3.2. Drp1 Damaged Mitochondria through the Overproduction of Reactive Oxygen Species (ROS) and Caused Energy Deficiency, Which Inhibited Proliferation of GH3 Cells

**Figure 2:**
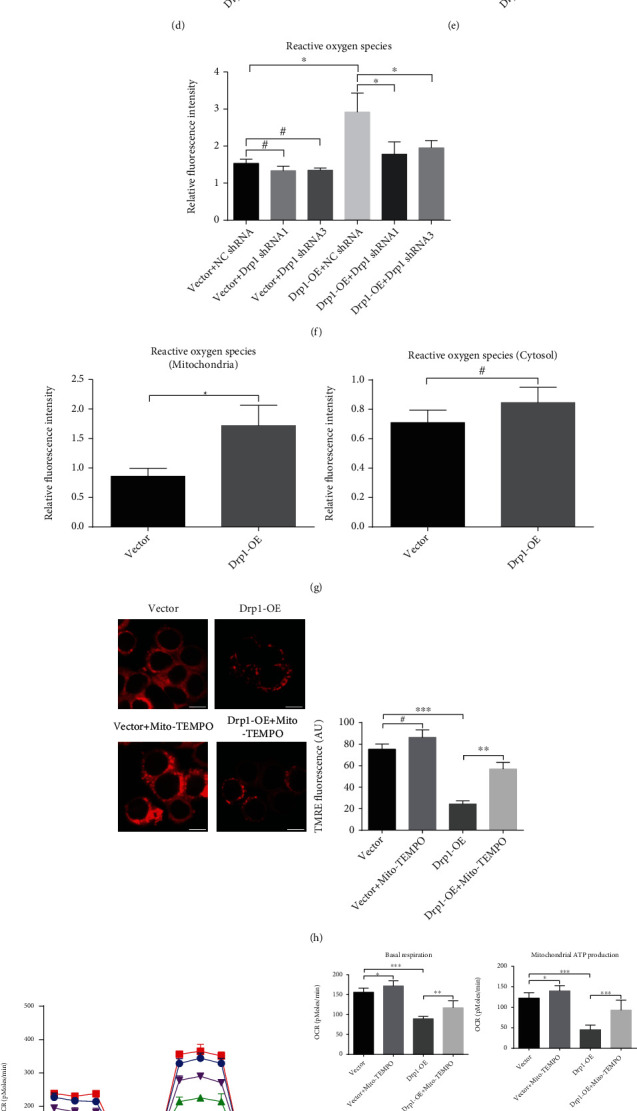
Drp1 damaged mitochondria through the overproduction of reactive oxygen species (ROS) and caused energy deficiency, which inhibited proliferation of GH3 cells. (a) Vector and Drp1-OE GH3 cells were incubated with/without 10 *μ*M Mdivi-1 for 48 h. Mitochondrial morphology was observed by transmission electron microscope (TEM) in each group (*n* = 9). Scale bar, 0.5 *μ*m. (b) Mitochondrial membrane potential (MMP) of GH3 cells was detected by using TMRE and observed by fluorescence microscope in the above four groups. Scale bar, 10 *μ*m. Fluorescence intensity was analyzed by ImageJ (*n* = 3, ± SEM). (c) Oxygen consumption rate (OCR) was detected in the above four groups (left). Four key parameters (basal respiration, mitochondrial ATP production, maximal respiration, spare respiratory capacity) of OCR were statistical analyzed (right, *n* = 9, ± SEM). (d) Total ATP production was measured in the above four groups (*n* = 3, ± SEM). (e) Intracellular ROS levels in the above four groups were detected by flow cytometry (*n* = 3, ±SEM). (f) Vector and Drp1-OE GH3 cells were infected with NC shRNA or Drp1 shRNA (Drp1 shRNA1 or Drp1 shRNA3). Then, intracellular ROS levels were detected in each group (*n* = 3, ±SEM). (g) Mitochondrial ROS levels were detected by flow cytometry in isolated mitochondria of the vector and Drp1-OE groups (left, *n* = 3, ±SEM). The cytosolic ROS levels were calculated from the data of intracellular and mitochondrial ROS (right, *n* = 3, ±SEM). (h) Vector and Drp1-OE GH3 cells were incubated with/without 200 *μ*M Mito-TEMPO for 48 h. MMP was detected by using TMRE and observed by fluorescence microscope in the above four groups. Scale bar, 10 *μ*m. Fluorescence intensity was analyzed by ImageJ (*n* = 3, ± SEM). (i) OCR was determined in the above four groups (left). Four key parameters of OCR were statistical analyzed (right, *n* = 9, ± SEM). (j) Total ATP production was measured in the above four groups (*n* = 3, ± SEM). (k) Vector and Drp1-OE GH3 cells were incubated with/without 200 *μ*M Mito-TEMPO for 0, 24, 48, or 72 h, and cell proliferation was assessed by a CCK-8 assay (*n* = 6, ± SEM) at different time points. (l) A simple schematic showing the results in Figure 2. An unpaired *t*-test was used to assess statistical significance. ^∗^*P* < 0.05; ^∗∗^*P* < 0.01; ^∗∗∗^*P* < 0.001; #, not significant.

Then, we assessed the effects of Drp1-OE treatment on mitochondrial structure and function of GH3 cells. By use of transmission electron microscopy (TEM), we observed that Drp1-OE GH3 cells exhibited obvious structural damage, such as mitochondrial swelling, vacuole formation, and decreased or absent cristae folds. Simultaneously, inhibition of Drp1 by Mdivi-1 partially repaired mitochondrial damage (Figure 2(a)). Subsequently, mitochondrial membrane potential (MMP) of GH3 cells was detected by using TMRE. We found that Drp1-OE treatment caused a significant decline in fluorescence intensity of GH3 cells, which indicated MMP loss. In contrast, Mdivi-1 treatment obviously reversed the fluorescence intensity in Drp1-OE group cells, which indicated MMP recovery (Figure 2(b)). Moreover, we compared the mitochondrial respiration rate (OCR), which represents the oxidative phosphorylation (OXPHOS) level, in the control and Drp1-OE group cells. As shown in Figure 2(c), the four key parameters (basal respiration, maximal respiration, mitochondrial ATP production, and spare respiratory capacity) of the OCR were significantly reduced by Drp1-OE treatment and could be rescued by Mdivi-1. All these changes related to mitochondrial function indicated a decline in mitochondrial activity and OXPHOS, which is essential for ATP synthesis. Tumor cell proliferation is a highly ATP-dependent cellular process, and there is a proportional relationship between the rate of tumor cell proliferation and the rate of ATP supply [10, 34]. Consequently, intracellular total ATP production was assessed. The results showed that Drp1-OE led to a significant decline in total ATP, which could be reversed by using Mdivi-1 (Figure 2(d)). To establish whether OXPHOS decline was responsible for the decrease of total ATP production in GH3 cells, we measured the extracellular acidification rate (ECAR), which represents the glycolysis level, in the control and Drp1-OE group cells. We found that the regulation to Drp1 did not obviously change the key parameters of the ECAR (Figure S6(a)), which suggested that glycolysis did not play a compensatory role in Drp1-induced energy deficiency. The above results demonstrated that Drp1-mediated proliferative inhibition was due to the decline in intracellular ATP production, which was generated by the suppressed OXPHOS. Oxidative stress is closely related to mitochondrial function [35–38]. Thus, we detected the ROS levels in GH3 cells after Drp1-OE treatment. Fluorescence intensity analysis showed that the ROS level in the Drp1-OE group was obviously increased compared with that in the control group but was partially decreased in the Mdivi-1 rescue group (Figure 2(e)). The Drp1 shRNA transfection also obviously declined the ROS level in the Drp1-OE group (Figure 2(f)). To establish whether mitochondria were responsible for the change of ROS production in GH3 cells, we measured the ROS levels in mitochondria and cytosol, respectively. Fluorescence intensity analysis showed that the ROS level in the Drp1-OE group was obviously increased compared with that in the control group in isolated mitochondria (Figure 2(g), left). However, there were no significant differences in cytosolic ROS between the two groups (Figure 2(g), right). These results suggested that the increased ROS level in GH3 cells after Drp1-OE treatment was mainly due to mitochondria. A mitochondrial-specific ROS scavenger, Mito-TEMPO, was applied to investigate this possibility. We found that the decreased MMP and OCR in the Drp1-OE treatment groups were all rescued by using Mito-TEMPO (Figures 2(h) and 2(i)). These results suggested that Drp1-induced overproduction of ROS led to mitochondrial oxidative damage, which inhibited OXPHOS in GH3 cells. Subsequently, intracellular total ATP production and cell proliferative capacity were assessed. The results showed that Drp1-OE led to a significant decline in total ATP, which could be reversed by using Mito-TEMPO (Figure 2(j)). CCK-8 assays showed that Drp-OE-induced cell proliferative suppression was rescued by Mito-TEMPO treatment (Figure 2(k)). Mito-TEMPO did not obviously change the key parameters of the ECAR (Figure S6(b)), which inferred that glycolysis did not play a compensatory role in ROS-mediated energy deficiency. Taken together, the above results suggested that Drp1 damaged mitochondria through the overproduction of ROS and caused energy deficiency, which inhibited proliferation of GH3 cells. A simple schematic of this process was presented (Figure 2(l)).

### 3.3. Drp1 Promoted the Cytochrome c Release from Damaged Mitochondria and Induced Apoptosis of GH3 Cells by Activating Downstream Proapoptotic Proteins

**Figure 3:**
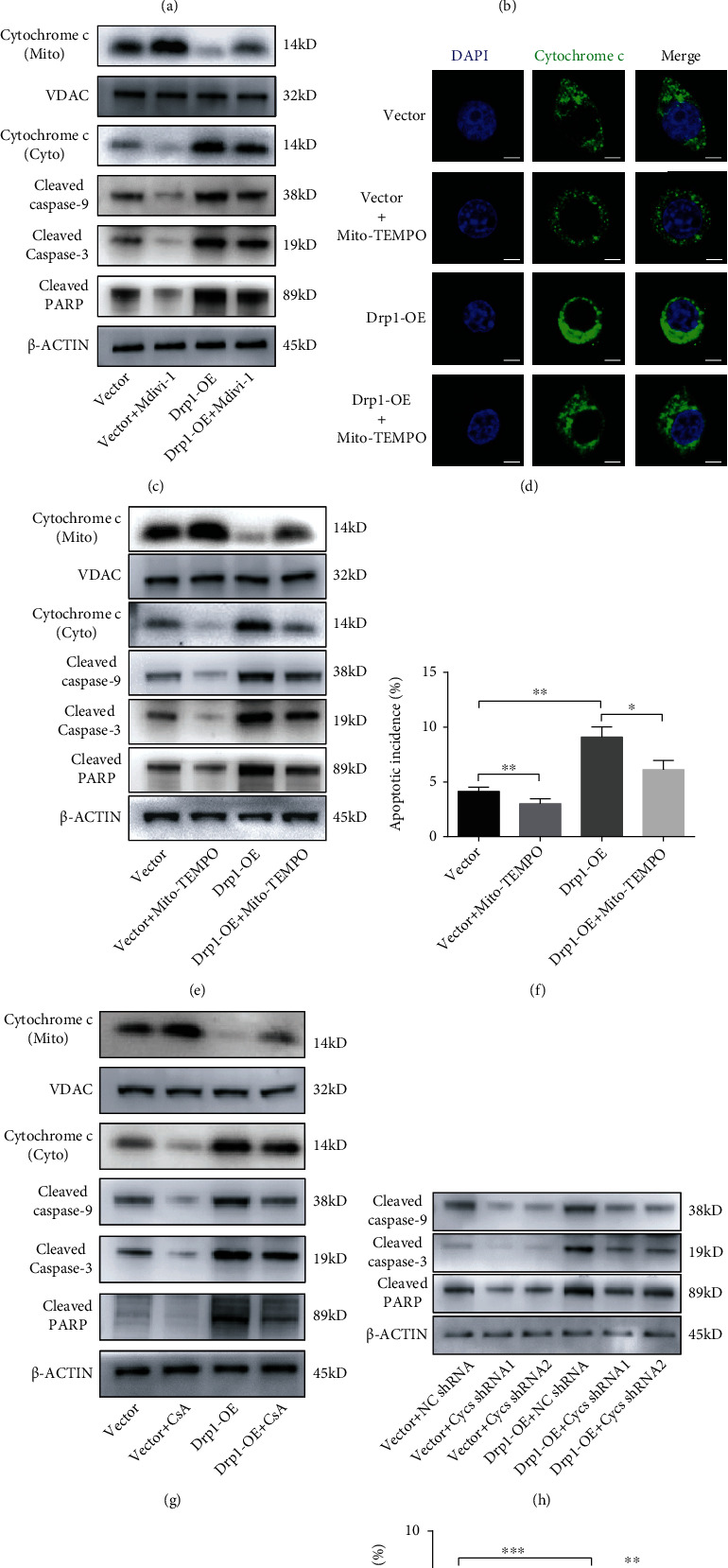
Drp1 promoted the cytochrome c release from damaged mitochondria and induced apoptosis of GH3 cells by activating downstream proapoptotic proteins. (a) A simple schematic showing the modulation of the Drp1-induced apoptotic pathway at different levels. Mdivi-1 inhibits the activity of Drp1. Mito-TEMPO is a mitochondrial specific ROS scavenger. CsA inhibited the release of cytochrome c from the mitochondria by preventing the formation of mPTP. Cytochrome c gene (Cycs) shRNAs were used to knock down Cycs expression. (b) Vector and Drp1-OE GH3 cells were incubated with/without 10 *μ*M Mdivi-1 for 48 h. Representative immunostaining of cytochrome c was observed by laser confocal microscopy in each group. Scale bar, 7.5 *μ*m. (c) Protein expression levels of mitochondrial (mito) cytochrome c, cytosolic (cyto) cytochrome c, and downstream proapoptotic proteins in each groups were assessed by western blotting. (d) Vector and Drp1-OE GH3 cells were incubated with/without 200 *μ*M Mito-TEMPO for 48 h. Immunostaining of cytochrome c was observed by laser confocal microscopy in each group. Scale bar, 7.5 *μ*m. (e) Expression levels of cytochrome c and downstream proapoptotic proteins in each group were assessed by western blotting. (f) Vector and Drp1-OE GH3 cells were incubated with/without 200 *μ*M Mito-TEMPO for 48 h. Cell apoptosis was measured by flow cytometry (*n* = 3, ± SEM). (g) Vector and Drp1-OE GH3 cells were incubated with/without 1 *μ*M CsA for 48 h. Expression levels of cytochrome c and downstream proapoptotic proteins were assessed by western blotting. (h) Vector and Drp1-OE GH3 cells were infected with NC shRNA or Cycs shRNA (Cycs shRNA1 or Cycs shRNA2). Expression levels of downstream proapoptotic proteins were examined by western blotting at 48 h. (i) Vector and Drp1-OE GH3 cells were incubated with/without 1 *μ*M CsA for 48 h. Cell apoptosis at 48 h was measured by flow cytometry (*n* = 3, ± SEM). (j) Vector and Drp1-OE GH3 cells were infected with NC shRNA or Cycs shRNA (Cycs shRNA1 or Cycs shRNA2). Cell apoptosis at 48 h was measured by flow cytometry (*n* = 3, ± SEM). An unpaired *t*-test was used to assess statistical significance. ^∗^*P* < 0.05; ^∗∗^*P* < 0.01; ^∗∗∗^*P* < 0.001; #, not significant.

Overproduction of ROS is tightly related to the mitochondrial apoptotic pathway [39]. A simple schematic of this signaling pathway and modulation to the pathway at different levels was designed and presented (Figure 3(a)). We investigated the cellular distribution of cytochrome c in GH3 cells by using immunostaining. Cytochrome c staining of control group cells was concentrated in mitochondria, while that of Drp1-OE cells diffused into the cytoplasm, and Mdivi-1 reversed this process induced by Drp1 (Figure 3(b)). Simultaneously, western blotting confirmed that the expression of cytochrome c in mitochondria decreased and that in the cytoplasm increased under Drp1-OE treatment, which could be partially reversed by Mdivi-1, suggesting that cytochrome c was translocated from mitochondria to the cytoplasm. Following the abnormal distribution of cytochrome c within the cytoplasm, the expression levels of the proapoptotic proteins cleaved caspase-9, cleaved caspase-3, and cleaved PARP were upregulated, which indicated the activation of the mitochondrial apoptotic pathway (Figure 3(c); Figure S2(c)). Then, the above proapoptotic proteins were detected after Mito-TEMPO (a special ROS scavenger) treatment in GH3 cells. Both immunostaining and western blotting results showed that cytochrome c release from mitochondria was obviously inhibited by using Mito-TEMPO, while the levels of downstream proteins were also suppressed (Figures 3(d) and 3(e), Figure S2(d)). The flow cytometry assay showed that Drp-OE-induced cell apoptosis was repressed by Mito-TEMPO treatment (Figure 3(f), Figure S4(c)). Cyclosporin A (CsA), a potent and widely used inhibitor of the mitochondrial permeability transition pore (mPTP), can prevent the release of cytochrome c from mitochondria [40, 41]. Western blotting results showed that the level of cytochrome c release was obviously suppressed in both the control and Drp1-OE groups after CsA treatment, as well as the levels of downstream proapoptotic proteins (Figure 3(g), Figure S2(e)). The flow cytometry assay showed that after treatment with CsA, the promoting effect of Drp1-OE on cell apoptosis was suppressed (Figure 3(i), Figure S4(d)). To further confirm the effect of cytochrome c on cell apoptosis, Cycs shRNAs were used to knock down the expression of cytochrome c. The transfection efficiency was confirmed by RT-qPCR (Figure S3(b)). We observed that after treatment with Cycs shRNAs, the levels of downstream proapoptotic proteins decreased obviously in the Drp1-OE group (Figure 3(h), Figure S2(f)). The flow cytometry assays showed that downregulation of cytochrome c expression alleviated the promoting effect of Drp-OE on cell apoptosis (Figure 3(j), Figure S4(e)). Taken together, the above results suggested that the mitochondrial oxidative damage caused by Drp1 promoted the apoptosis of GH3 cells through the mitochondrial apoptotic pathway.

### 3.4. Drp1-Induced Energy Stress Enhanced Cell Protective Autophagy by Activating the AMPK-ULK1 Pathway

**Figure 4:**
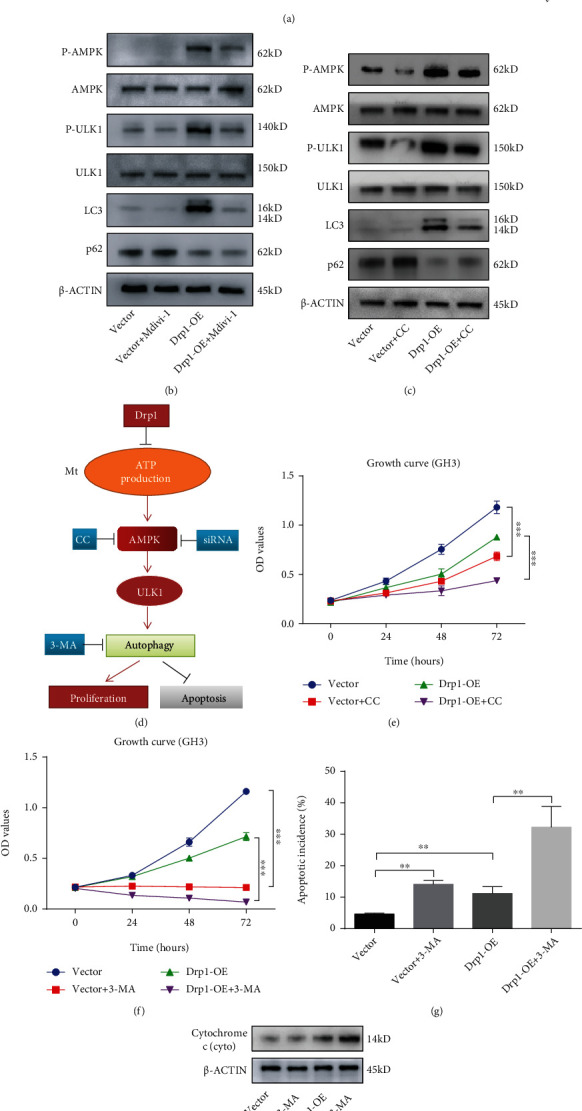
Drp1-induced energy stress enhanced cell protective autophagy by activating the AMPK-ULK1 pathway. (a) Vector and Drp1-OE GH3 cells were incubated with/without 10 *μ*M Mdivi-1 for 48 h. Autophagosomes were observed by TEM in each group (left). Statistical analysis of the autophagosomes (right). Scale bar, 0.5 *μ*m (*n* = 9, ± SEM). (b–c) Vector and Drp1-OE GH3 cells were pretreated with/without Mdivi-1 (10 *μ*M) or Compound C (CC, 2.5 *μ*M) for 48 h. The total cell lysates were subjected to western blotting, and the protein expression levels of AMPK-ULK1 autophagy pathway were detected, respectively. (d) A simple schematic showing the modulation of the Drp1-induced autophagic pathway at different levels. (e) Vector and Drp1-OE GH3 cells were incubated with/without 2.5 *μ*M CC for 0, 24, 48, or 72 h, and cell proliferation at different time points was assessed by a CCK-8 assay (*n* = 6, ± SEM). (f) Vector and Drp1-OE GH3 cells were pretreated with/without 3-MA (10 mM) for 0, 24, 48, or 72 h, and cell proliferation at different time points was assessed by a CCK-8 assay (*n* = 6, ± SEM). (g) Cell apoptosis at 48 h was measured by flow cytometry (*n* = 3, ± SEM). (h) Vector and Drp1-OE GH3 cells were incubated with/without 3-MA (10 mM) for 48 h. Cytochrome c protein level was detected by western blotting. An unpaired *t*-test was used to assess statistical significance. ^∗^*P* < 0.05; ^∗∗^*P* < 0.01; ^∗∗∗^*P* < 0.001.

While observing the morphology of mitochondria with TEM, we found a significantly increased number of autophagosomes in the Drp1-OE group, which could be reversed by Mdivi-1 in GH3 cells (Figure 4(a)). In previous experiments, we confirmed that Drp1-OE treatment reduced total ATP production of GH3 cells. It has been reported that energy stress can activate cellular autophagy via AMPK-ULK1 pathway [42–44]. Western blotting results showed that the protein expression levels of phosphorylated AMPK and ULK1 were significantly upregulated in Drp1-OE GH3 cells and coupled with increased expression of LC3 protein and decreased expression of p62 protein, which could be partially reversed by Mdivi-1 (Figure 4(b), Figure S2(g)). Moreover, AMPK inactivation by Compound C (CC, an AMPK inhibitor) treatment or siRNA-mediated AMPK knockdown significantly alleviated the Drp1-induced activation of AMPK-ULK1 pathway (Figure 4(c), Figure S2(h); Figure S7(a); Figure S3(c)-(d)). These results suggested that Drp1 promoted autophagy processes by activating the AMPK-ULK1 pathway. Furthermore, we investigated the effect of autophagy on cell proliferation and apoptosis. A simple schematic of the signaling pathway and modulation to the pathway at different levels was designed and presented (Figure 4(d)). First, we detected that Compound C or AMPK siRNAs not only attenuated the enhanced autophagy level mediated by Drp1 but also suppressed cell proliferation in both the control and Drp1-OE groups (Figure 4(e), Figure S7(b)). Then, 3-MA (a widely used inhibitor of autophagy) was applied to inhibit autophagy. We observed that 3-MA treatment dramatically suppressed the proliferation of GH3 cells in both the control and Drp1-OE groups, as shown by CCK-8 assays (Figure 4(f)). Simultaneously, the flow cytometry assay showed that Drp-OE-induced cell apoptosis was promoted by 3-MA treatment (Figure 4(g), Figure S4(f)). In addition, we found that 3-MA treatment increased the level of cytochrome c in the cytoplasm of Drp1-OE group cells (Figure 4(h), Figure S2(i)), which suggested that autophagy could impact the mitochondrial apoptotic pathway by degrading cytochrome c. These results indicated that autophagy might play a protective role in the growth of PA. Collectively, our data indicated that Drp1 generated energy stress in GH3 cells, which enhanced autophagy levels by activating the AMPK-ULK1 pathway to protect cell survival.

### 3.5. Drp1 Regulated PA Growth In Vivo

**Figure 5:**
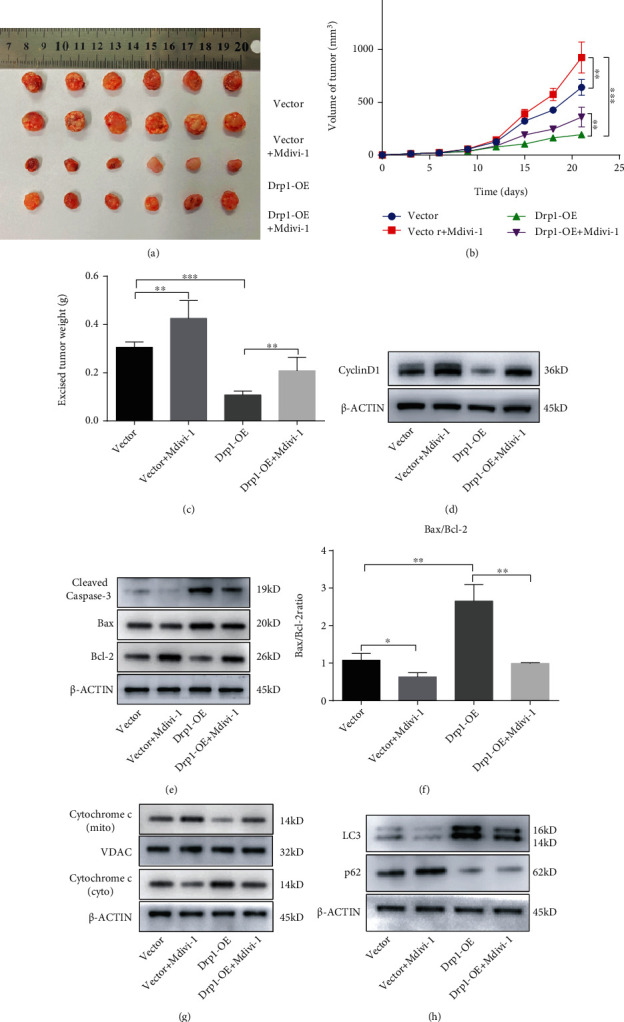
Drp1 regulated PA growth in vivo. (a) Excised tumors in different treatment groups are shown. (b) Growth curve showing the changes in the tumor volume in mice after different treatments. (c) Weight of the excised tumors in each group. (d) Protein expression level of Cyclin D1 in excised tumors was detected by western blotting. (e) Protein expression levels of cleaved caspase-3, Bax, and Bcl-2 in excised tumors were detected by western blotting. (f) Bax/Bcl-2 ratio was calculated by relative protein expression. (g) Protein expression levels of cytochrome c (mito) and cytochrome c (cyto) in excised tumors were examined by western blotting. (h) Protein expression levels of LC3 and p62 in excised tumors were detected by western blotting. An unpaired *t*-test was used to assess statistical significance. ^∗^*P* < 0.05; ^∗∗^*P* < 0.01; ^∗∗∗^*P* < 0.001.

To further evaluate the effects of Drp1 on PA growth in vivo, a PA xenograft model was generated by subcutaneous injection of either control or Drp1-OE GH3 cells into nude mice. The mice were randomly divided into four groups. Nine days after injection, the mice were administered PBS or Mdivi-1 (50 mg/kg) daily for 3 weeks. We found that Drp1-OE significantly inhibited tumor growth, while Mdivi-1 obviously promoted tumor growth in vivo (Figures 5(a)–5(c)). Mdivi-1 treatment did not significantly alter mouse body weight (Figure S8). Subsequently, western blotting was performed to detect the expression levels of markers of cell cycle, apoptosis, and autophagy in PA xenograft tumor samples, respectively. The results indicated that Drp1-OE treatment obviously downregulated expression of Cyclin D1 (Figure 5(d), Figure S2(j)) and upregulated the expression of caspase-3 and the ratio of Bax/Bcl-2 (Figures 5(e) and 5(f), Figure S2(k)), which prompted that Drp1 could suppressed the cell proliferation and promoted the cell apoptosis in vivo. Besides, Drp1-OE treatment promoted the release of cytochrome c from mitochondria to cytosol (Figure 5(g), Figure S2(l)), which suggested that Drp1 might activate the mitochondrial apoptotic pathway in vivo. Moreover, we found that the LC3 protein level was upregulated and the p62 level was downregulated after Drp1-OE treatment (Figure 5(h), Figure S2(m)), which demonstrated that Drp1 activated cell autophagy in vivo. In sum, the above results proposed that Drp1 could affect PA growth by inhibiting cell proliferation and promoting apoptosis and autophagy in vivo.

## 4. Discussion

Few attempts have been made to elucidate the potential role of mitochondrial metabolism and key regulatory molecules in the progression of PA. We found that upregulated Drp1 expression was correlated with lower proliferative activity of human PA samples and that Drp1 overexpression not only inhibited PA cell proliferation but also promoted PA cell apoptosis in vitro and in vivo. Subsequently, we elucidated that overexpressed Drp1 damaged mitochondria through the overproduction of ROS and caused energy deficiency, which inhibited proliferation of PA cells. Furthermore, we confirmed that the oxidative damage induced by Drp1 promoted apoptosis of PA cells by activating the mitochondrial apoptotic pathway. In addition, we observed that Drp1 overexpression enhanced autophagy levels by activating the AMPK-ULK1 pathway in PA cells and demonstrated that autophagy might play a protective role in PA growth. Our study is the first showing that Drp1 can affect the progression of PA by directly regulating mitochondrial metabolism, which has provided new insight into the mechanism of PA progression.

As a member of the dynamin superfamily of GTPases, Drp1 mediates mitochondrial fission and affects mitochondrial morphology in mammalian cells [15–17], which permits renewal and redistribution inside the cells [18, 19]. In the previous study, Drp1 has been shown to affect the progression of many tumor types. Drp1 promotes the progression of brain tumor-initiating cells by regulating mitochondrial dynamics [20]. Phosphorylation of Drp1 at the Ser616 site significantly enhances the Warburg effect of ovarian cancer cells by promoting mitochondrial fission, which inhibits mitochondrial oxidative phosphorylation [21]. Silencing Drp1 causes mitochondrial elongation and significantly suppresses the metastatic abilities of breast cancer cells [22]. Increased Drp1 upregulation-mediated mitochondrial fission and subsequently enhanced aerobic glycolysis are involved in promoting growth and metastasis by Drp1 in pancreatic cancer cells [23]. The imbalance of mitochondrial homeostasis mediated by Drp1 results in dysfunction of endometrial cancer cells [24]. In our study, we found that Drp1 expression was higher in LI-PA samples than in HI-PA samples, and overexpressed Drp1 significantly inhibited the growth of PA cells. These results suggest that upregulated expression of Drp1 may be one of the internal reasons why LI-PA can maintain the low proliferative activity and that Drp1 may be a promising target for therapies in studies of PA.

Metabolic processes may result in an increase in the production of ROS [35], which is a vital metabolic substance in cellular oxidative stress. The increase of cellular ROS may own multiple sources. Mitochondria are the main source of them. The maintenance of a cell proliferation rhythm and cell survival depends on the redox homeostasis between ROS production and elimination, which occurs in the process of mitochondrial fission and fusion [45]. Physiological ROS levels contribute to the regulation of mitochondrial dynamics [36]. When the production of ROS exceeds the cellular antioxidant capacity, oxidative stress and oxidative damage to biomolecules (proteins, DNA, and RNA) may take place and promote the tumor progression [46]. Oxidative stress and mitochondrial damage play a critical role in PA progression [8, 47]. Our data indicated that Drp1-mediated overproduction of ROS disrupted redox homeostasis, which prevented proper ROS elimination, resulting in mitochondrial oxidative damage in PA cells. Mito-TEMPO has been recently reported as a mitochondria-targeted antioxidant with low toxicity that effectively alleviate mitochondrial ROS-induced oxidative stress [48]. In our experiments, we found that mitochondria were the main source of the excess ROS production in the Drp1-OE group cells and that Mito-TEMPO effectively repaired Drp1-induced mitochondrial dysfunction. These results indicate that mitochondrial ROS plays a key role in the Drp1-induced mitochondrial injury.

Energy homeostasis is pivotal for cell fate and metabolic regulation. Tumor cell proliferation is a highly ATP-dependent cellular process, and there is a proportional relationship between the rate of tumor cell proliferation and the rate of ATP supply [10, 34]. In our findings, the mitochondrial dysfunction caused by Drp1 led to a marked decline in total ATP production, which suggests that the inhibited proliferation of PA cells is due to the Drp1-mediated energy efficiency. OXPHOS and glycolysis are two major ways that tumor cells use glucose for ATP production. We observed that the decreased OXPHOS induced by Drp1 also led to a significant decrease in total ATP production. However, glycolysis did not obviously compensate for the energy deficiency. These results suggest that PA cells may have a more sufficient oxygen supply and a higher level of OXPHOS, which plays a crucial role in the energy supply to PA proliferation.

Overproduction of ROS is closely related to the mitochondrial apoptotic pathway [39]. The mitochondrial apoptotic pathway intrinsically regulates cell apoptotic events through mitochondrial membrane permeabilization and subsequent release of proapoptotic proteins [49]. Cytochrome c is an important proapoptotic protein that is also a key component of the OXPHOS system [50, 51]. The release of cytochrome c from mitochondria into the cytosol is one of the major events in mitochondrial apoptotic pathways. Cytosolic cytochrome c induces caspase-9-dependent activation of caspase-3 and cleavage of PARP DNA reparatory protein [52]. Studies have shown that mitochondrial fission promotes mitochondrial membrane depolarization, cytochrome c release, and apoptosis [53–56]. In PA cell lines and xenograft model experiments, we found that oxidative stress mediated by Drp1 promoted cytochrome c release from injured mitochondria into the cytosol, which induced cell apoptosis by upregulating downstream proapoptotic molecules. Our data emphasize the core role of mitochondria in the regulation of the programmed cell death in PA.

As a ubiquitous sensor of the cellular energy status, AMP-activated protein kinase (AMPK) is activated in response to energy stress and plays a major role in maintaining metabolic homeostasis [57, 58]. When cellular energy is low or depleted, AMPK directly phosphorylates the autophagy-initiating kinase ULK1 and thereby activates autophagy [44]. Autophagy is a catabolic process that plays a housekeeping role in eliminating protein aggregates and malfunctioning organelles and is responsible for the maintenance of normal cellular homeostasis in response to energy stress [59, 60]. There is a clear interconnection between mitochondria and autophagy. Autophagy can effectively segregate damaged mitochondria from a healthy network in cells, which facilitates their degradation and recycles degraded mitochondrial components [60–63]. In addition, mitochondria supply membranes for the biogenesis of autophagosomes during cell starvation [64]. Our previous data have shown that Drp1-OE treatment not only led to a decline in total ATP production mediated by OXPHOS inhibition but also activated autophagy through the AMPK-ULK1 pathway in PA cells. These results prompted that Drp1 activated autophagy through the mitochondria-mediated energy stress in PA cells.

Autophagy has been identified as a survival mechanism across several tumor types [65–68]. The association between tumor cell survival and autophagy can be partly explained by the role of autophagy in protecting cells from programmed cell death [69]. Severe defects in autophagy or mitophagy have been associated with decreased tumor progression in multiple models of oncogenesis [70–73]. Previous reports have proven that autophagy induced by AMPK contributes to neuroprotection [74, 75]. This view was supported by our findings that Compound C or AMPK siRNAs could markedly suppress the proliferative capacity of PA cells. However, the effect of autophagy on the ability of tumor cells to undergo apoptosis is not always protective but sometimes is proapoptotic [76]. The mechanisms underlying the opposing roles in tumors are related to the degradation of different proapoptotic or antiapoptotic regulators by autophagy [76, 77]. 3-Methyladenine (3-MA) is a widely used inhibitor of autophagy through its inhibitory effect on class III PI3K [78]. Our data showed that 3-MA caused a dramatic decline in the proliferation of PA cells and led to extensive programmed cell death. Moreover, we observed that 3-MA promoted the cytochrome c release in Drp1-OE group cells. These results imply that autophagy plays a protective role in the progression of PA and that cytochrome c may be one of the key proapoptotic regulators degraded in this process.

**Figure 6:**
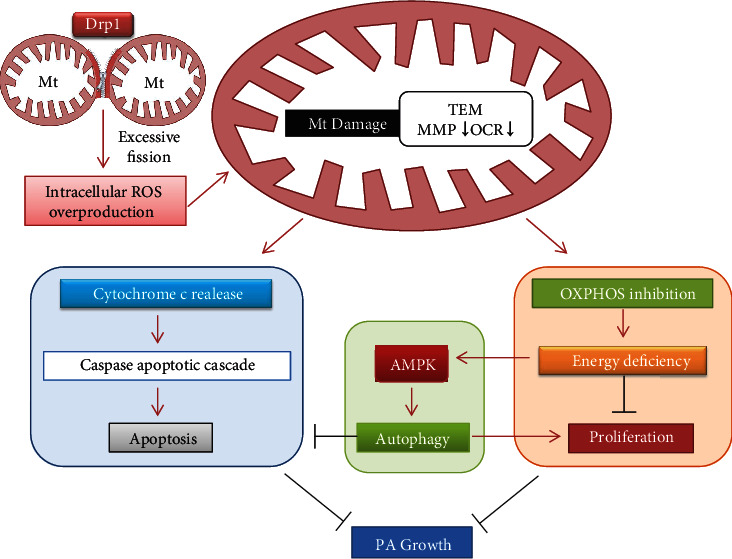
Drp1-mediated mitochondrial metabolic dysfunction inhibited PA growth. A proposed scheme illustrates the process that Drp1-mediated ROS overproduction causes mitochondrial metabolic dysfunction, which lead to PA growth inhibition under the combined effect of cell proliferation, apoptosis, and autophagy.

In summary, we first revealed that Drp1 expression was dysregulated in PA, and upregulated expression of Drp1 could attenuate the proliferative activity of PA. By causing oxidative damage to mitochondria, Drp1 induced energy stress and programmed cell death in PA cells, which inhibited proliferation and promoted apoptosis, respectively. On the other hand, Drp1-mediated autophagy might play a protective role in the above process (Figure 6). Our findings suggested that selectively targeting Drp1 might be a potential therapy for PA patients. However, the results of this study should be verified through further studies and clinical investigations.

## 5. Conclusions

In conclusion, our study showed that mitochondrial metabolic reprogramming also existed in PA. By regulating the generation or distribution of the metabolites (ROS, ATP, cytochrome c) in mitochondria, Drp1 markedly affected the proliferation, apoptosis, and autophagy of tumor cells, inhibiting the growth of PA. Taken together, our study provides new insight into the mechanism underlying PA progression and provides molecular mechanistic arguments for targeting mitochondrial metabolism in PA treatment.

## Data Availability

The data used to support the findings of this study are available from the corresponding author on reasonable request.

## Supplementary Materials

Supplementary Materials Table S1: clinical characteristics of 20 human surgical PA samples. Table S2: primer list for RT-qPCR. Table S3: target sequences of shRNAs and siRNAs. Figure S1: schematic diagrams of OCR and ECAR, which represent for the capacity of OXPHOS and glycolysis, respectively. (a) The curve of OCR demonstrates the key parameters of mitochondrial function: basal respiration, maximal respiration, mitochondrial ATP production, and spare respiratory capacity. (b) The curve of ECAR demonstrates the key parameters of glycolytic function: basal glycolysis, maximal glycolysis, and spare glycolytic capacity. Figure S2: statistical analysis of the western blotting results for different treatments. (a) Statistical analysis of the western blotting results in Figure 1(c) (*n* = 3, ± SEM). (b) Statistical analysis of the western blotting results in Figure 1(k) (*n* = 3, ± SEM). (c) Statistical analysis of the western blotting results in Figure 3(c) (*n* = 3, ± SEM). (d) Statistical analysis of the western blotting results in Figure 3(e) (*n* = 3, ± SEM). (e) Statistical analysis of the western blotting results in Figure 3(g) (*n* = 3, ± SEM). (f) Statistical analysis of the western blotting results in Figure 3(h) (*n* = 3, ± SEM). (g) Statistical analysis of the western blotting results in Figure 4(b) (*n* = 3, ± SEM). (h) Statistical analysis of the western blotting results in Figure 4(c) (*n* = 3, ± SEM). (i) Statistical analysis of the western blotting results in Figure 4(h) (*n* = 3, ± SEM). (j) Statistical analysis of the western blotting results in Figure 5(d) (*n* = 3, ± SEM). (k) Statistical analysis of the western blotting results in Figure 5(e) (*n* = 3, ± SEM). (l) Statistical analysis of the western blotting results in Figure 5(g) (*n* = 3, ± SEM). (m) Statistical analysis of the western blotting results in Figure 5(h) (*n* = 3, ± SEM). An unpaired test was used to assess statistical significance. ∗*P* < 0.05; ∗∗*P* < 0.01; ∗∗∗*P* < 0.001; #, not significant. Figure S3: evaluation of shRNA transfection efficiency. (a) Drp1 mRNA level was measured in GH3 cells by RT-qPCR after transfection with shRNA. Drp1 shRNA1 and Drp1 shRNA3 were used for subsequent experiments, according to the expression level of Drp1 (*n* = 3, ± SEM). (b) Cytochrome c gene (Cycs) mRNA level was measured in GH3 cells by RT-qPCR after transfection with shRNA. Cycs shRNA1 and Cycs shRNA2 were used for subsequent experiments, according to the expression level of Cycs (*n* = 3, ± SEM). (c, d) AMPK*α*1 gene (Prkaa1) mRNA and AMPK*α*2 gene (Prkaa2) mRNA levels were measured in GH3 cells by RT-qPCR after transfection with siRNAs, respectively (*n* = 3, ± SEM). An unpaired test was used to assess statistical significance. ∗∗*P* < 0.01; ∗∗∗*P* < 0.001; #, not significant. Figure S4:flow cytometry plots of cell apoptosis for different treatments. (a) Flow cytometry plots of cell apoptosis in Figure 1(h). (b) Flow cytometry plots of cell apoptosis in Figure 1(i). (c) Flow cytometry plots of cell apoptosis in Figure 3(f). (d) Flow cytometry plots of cell apoptosis in Figure 3(i). (e) Flow cytometry plots of cell apoptosis in Figure 3(j). (f) Flow cytometry plots of cell apoptosis in Figure 4(g). Figure S5: protein expression of key molecules of ER stress-related unfolded protein response (UPR). Protein expression levels of p-IRE1, XBP1s, p-PERK, p-eIF2*α*, and ATF6 were detected by western blotting in the vector and Drp1-OE groups (*n* = 3, ± SEM). An unpaired *t*-test was used to assess statistical significance. #, not significant. Figure S6: Drp1 and ROS did not obviously alter the key parameters of extracellular acidification rate (ECAR). (a) Vector and Drp1-OE GH3 cells were incubated with/without 10 *μ*M Mdivi-1 for 48 h. ECAR was detected in the above four groups, respectively (left). The key parameters (basal glycolysis, maximal glycolysis, and spare glycolytic capacity) of ECAR were statistical analyzed (right, *n* = 9, ± SEM). (b) Vector and Drp1-OE GH3 cells were incubated with/without 200 *μ*M Mito-TEMPO for 48 h. ECAR was determined in the above four groups (left). The key parameters of ECAR were statistical analyzed (right, *n* = 9, ± SEM). An unpaired test was used to assess statistical significance. ∗*P* < 0.05; ∗∗*P* < 0.01; #, not significant. Figure S7: AMPK siRNAs alleviated the Drp1-induced activation of AMPK-ULK1 pathway and suppressed the proliferative capacity of PA cells. (a) Vector and Drp1-OE GH3 cells were pretreated with/without AMPK siRNAs (40 nM) for 24 h. The total cell lysates were subjected to western blotting, and the protein expression levels of AMPK-ULK1 autophagy pathway were detected, respectively (*n* = 3, ± SEM). (b) Vector and Drp1-OE GH3 cells were incubated with/without 40 nM AMPK siRNAs for 0, 24, 48, or 72 h, and cell proliferation at different time points was assessed by a CCK-8 assay (*n* = 6, ± SEM). An unpaired test was used to assess statistical significance. ∗*P* <0.05; ∗∗*P* <0.01; ∗∗∗*P* <0.001; #, not significant. Figure S8: body weight of mice in each group. Mdivi-1 did not significantly alter mice body weight (Vector vs. Vector + Mdivi-1, *P* = 0.313; Drp1-OE vs. Drp1-OE + Mdivi-1, *P* = 0.811). Statistical significance was determined by repeated measure analysis of variance. These analyses were performed using SPSS for Windows, version 13.0 (SPSS Inc., USA).
