# Supplementary material for: Drp1-Mediated Mitochondrial Metabolic Dysfunction Inhibits the Tumor Growth of Pituitary Adenomas
Source: Oxid Med Cell Longev. 2022 Mar 23;2022:5652586. doi: 10.1155/2022/5652586 (PMC8967574; doi:10.1155/2022/5652586)
Supplement: Supplementary Materials — Table S1: clinical characteristics of 20 human surgical PA samples. Table S2: primer list for RT-qPCR. Table S3: target sequences of shRNAs and siRNAs. Figure S1: schematic diagrams of OCR and ECAR, which represent for the capacity of OXPHOS and glycolysis, respectively. (a) The curve of OCR demonstrates the key parameters of mitochondrial function: basal respiration, maximal respiration, mitochondrial ATP production, and spare respiratory capacity. (b) The curve of ECAR demonstrates the key parameters of glycolytic function: basal glycolysis, maximal glycolysis, and spare glycolytic capacity. Figure S2: statistical analysis of the western blotting results for different treatments. (a) Statistical analysis of the western blotting results in Figure 1(c) (n = 3, ± SEM). (b) Statistical analysis of the western blotting results in Figure 1(k) (n = 3, ± SEM). (c) Statistical analysis of the western blotting results in Figure 3(c) (n = 3, ± SEM). (d) Statistical analysis of the western blotting results in Figure 3(e) (n = 3, ± SEM). (e) Statistical analysis of the western blotting results in Figure 3(g) (n = 3, ± SEM). (f) Statistical analysis of the western blotting results in Figure 3(h) (n = 3, ± SEM). (g) Statistical analysis of the western blotting results in Figure 4(b) (n = 3, ± SEM). (h) Statistical analysis of the western blotting results in Figure 4(c) (n = 3, ± SEM). (i) Statistical analysis of the western blotting results in Figure 4(h) (n = 3, ± SEM). (j) Statistical analysis of the western blotting results in Figure 5(d) (n = 3, ± SEM). (k) Statistical analysis of the western blotting results in Figure 5(e) (n = 3, ± SEM). (l) Statistical analysis of the western blotting results in Figure 5(g) (n = 3, ± SEM). (m) Statistical analysis of the western blotting results in Figure 5(h) (n = 3, ± SEM). An unpaired test was used to assess statistical significance. ∗P < 0.05; ∗∗P < 0.01; ∗∗∗P < 0.001; #, not significant. Figure S3: evaluation of shRNA transfection ef [file 5652586.f1.pdf]

Table S1: Clinical characteristics of 20 human surgical PA samples.

Case	Age [years]	Gender	Hormonal type	KI-67≥3%	P53
1	62	F	NFPA	-	-
2	54	M	NFPA	-	-
3	49	F	GH	-	-
4	26	M	GH	-	-
5	35	F	ACTH	-	-
6	38	M	ACTH	-	-
7	20	M	PRL	-	-
8	25	M	PRL	-	-
9	54	M	TSH	-	-
10	33	F	TSH	-	-
11	58	F	NFPA	+	+
12	64	M	NFPA	+	+
13	53	F	NFPA	+	+
14	54	M	NFPA	+	+
15	53	F	NFPA	+	+
16	52	M	NFPA	+	+
17	48	M	NFPA	+	+
18	42	F	GH	+	+
19	24	F	GH	+	+
20	51	F	GH	+	+

Table S2: Primer list for qPCR.

Gene name (Species)	Primer sequences
Drp1（human）	F: GAGATGGTGTTCAAGAACCAAC<br>R: CAATAACCTCACAATCTCGCTG
Drp1（rat）	F: GAGAACTACCTTCCGCTGTATCGC<br>R: CACCATCTCCAATTCCACCACCTG
Cycs (rat)	F: CTGCTGGATTCTCTTACACAGATGCC<br>R: TGCCCTTTCTCCCTTCTTCTTAATTCC
Prkaa1 (rat)	F: TTGCGTGTGCGAAGGAAGAACC<br>R: CCGATCTCTGTGGAGTAGCAGTCC
Prkaa2 (rat)	F: ATGATGAGGTGGTGGAGCAGAGG<br>R: GTTCTCGGCTGTGCTGGAATCG
β-actin (human)	F:CCTGGCACCCAGCACAAT<br>R:GGGCCGGACTCGTCATAC
β-actin (rat)	F:TGTCACCAACTGGGACGATA<br>R:GGGGTGTTGAAGGTCTCAAA

Table S3: Target sequences of shRNAs and siRNAs.

shRNA/siRNA name (Species)	Target sequences
Drp1 shRNA1 (rat)	GCGCTGATCCCGGTCATCAAT
Drp1 shRNA2 (rat)	GAGCTTCAAATCAGAGAACTT
Drp1 shRNA3 (rat)	GCGCAGAACTCTAGCTGTAAT
Cycs shRNA1 (rat)	GCCAACAAGAACAAAGGTATC
Cycs shRNA2 (rat)	AAAGTACATCCCTGGAACAAA
Cycs shRNA3 (rat)	ATGGTCTGTTTGGGCGGAAGA
AMPKα1 siRNA1 (rat)	CACGAGUUGACUGGACAUATT
AMPKα1 siRNA2 (rat)	GAAGACCCGUCUUAUAGUUTT
AMPKα1 siRNA3 (rat)	GGAUAGUAGGACUUACUUATT
AMPKα2 siRNA1 (rat)	GUGAAUUGUUCGACUACAUTT
AMPKα2 siRNA2 (rat)	CUGACUUCGGACUCUCUAATT
AMPKα2 siRNA3 (rat)	GCAUACCAUCUUCGAGUAATT

Figure S1

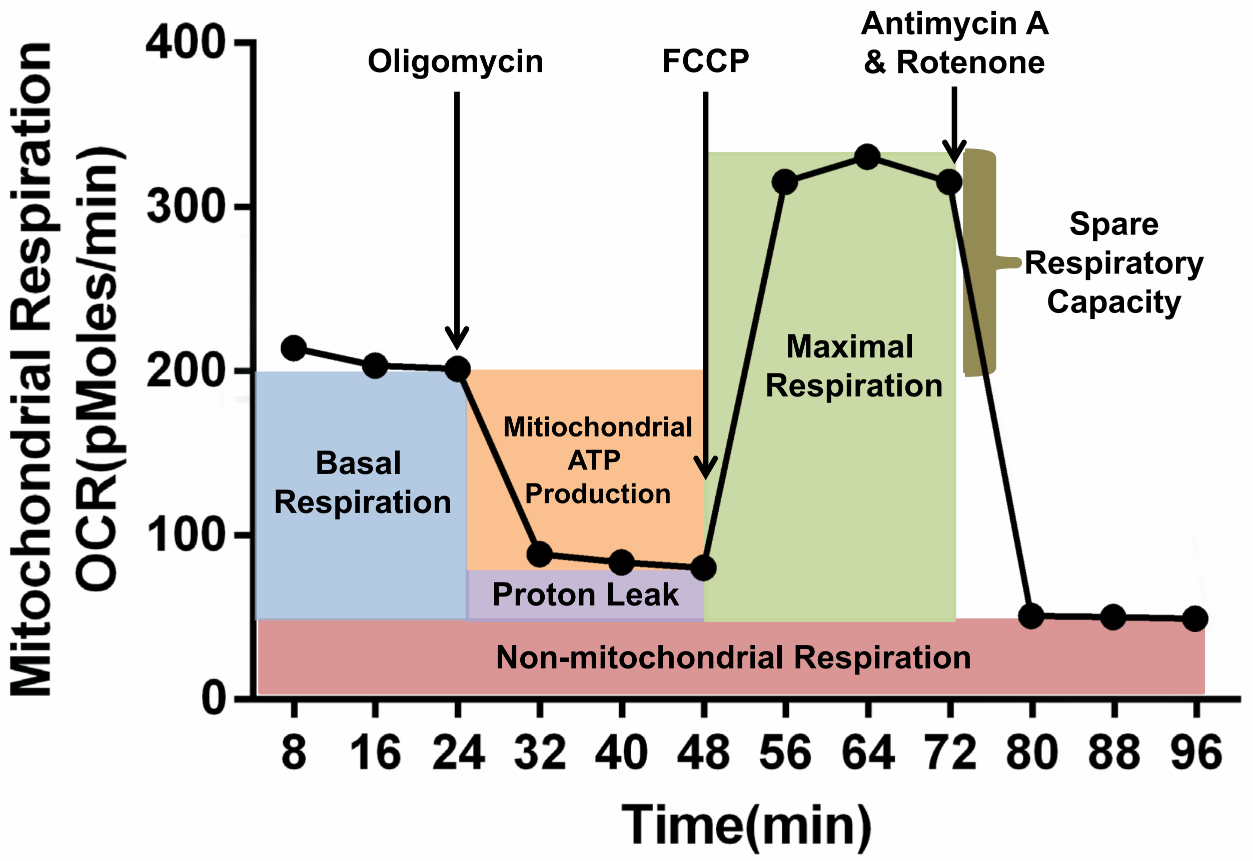
Mitochondrial Respiration
OCR(pMoles/min)
400
300
200
100
0
Oligomycin
FCCP
Antimycin A
& Rotenone
Spare
Respiratory
Capacity
Maximal
Respiration
Basal
Respiration
Mitiochondrial
ATP
Production
Proton Leak
Non-mitochondrial Respiration
8 16 24 32 40 48 56 64 72 80 88 96
Time(min)

(a)

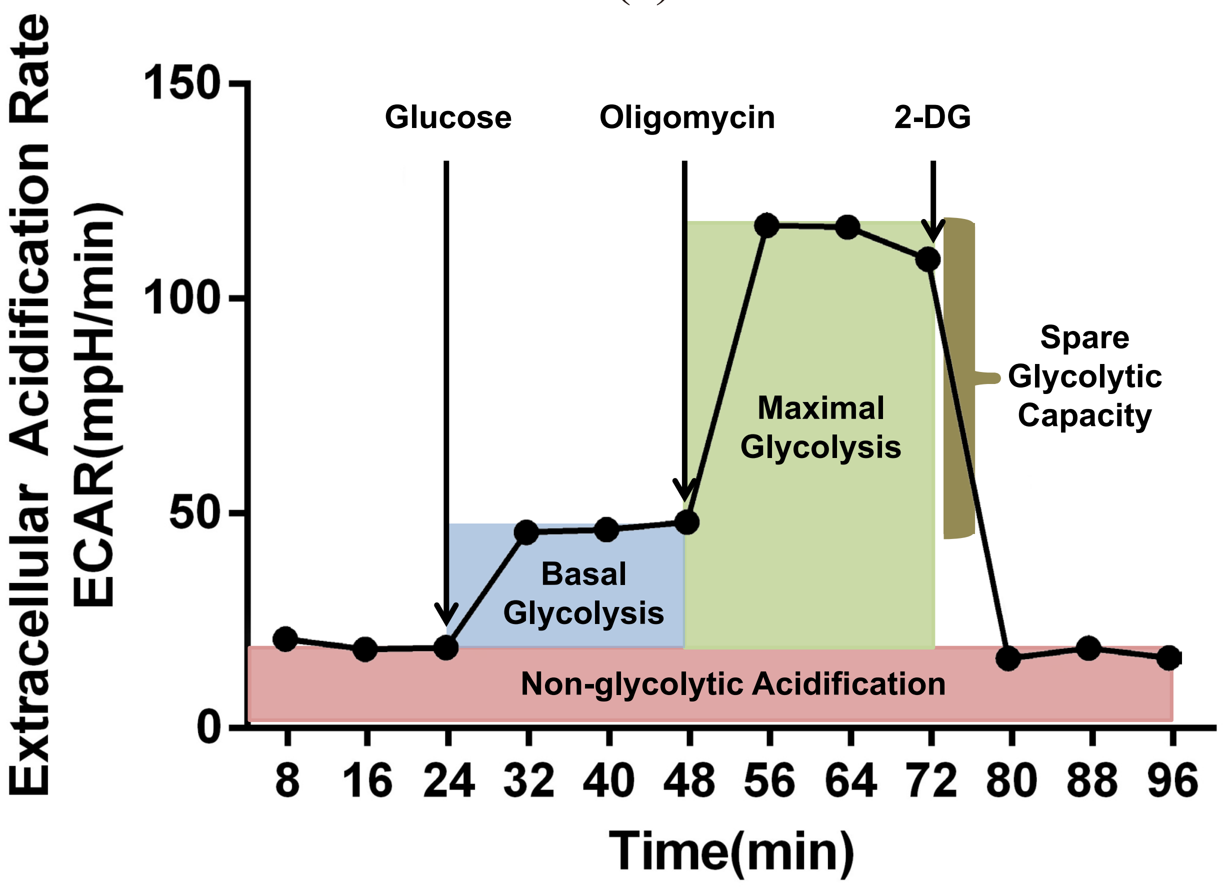
Extracellular Acidification Rate
ECAR(mpH/min)
150
100
50
0
Glucose
Oligomycin
2-DG
Spare
Glycolytic
Capacity
Maximal
Glycolysis
Basal
Glycolysis
Non-glycolytic Acidification
8 16 24 32 40 48 56 64 72 80 88 96
Time(min)

(b)

Figure S2

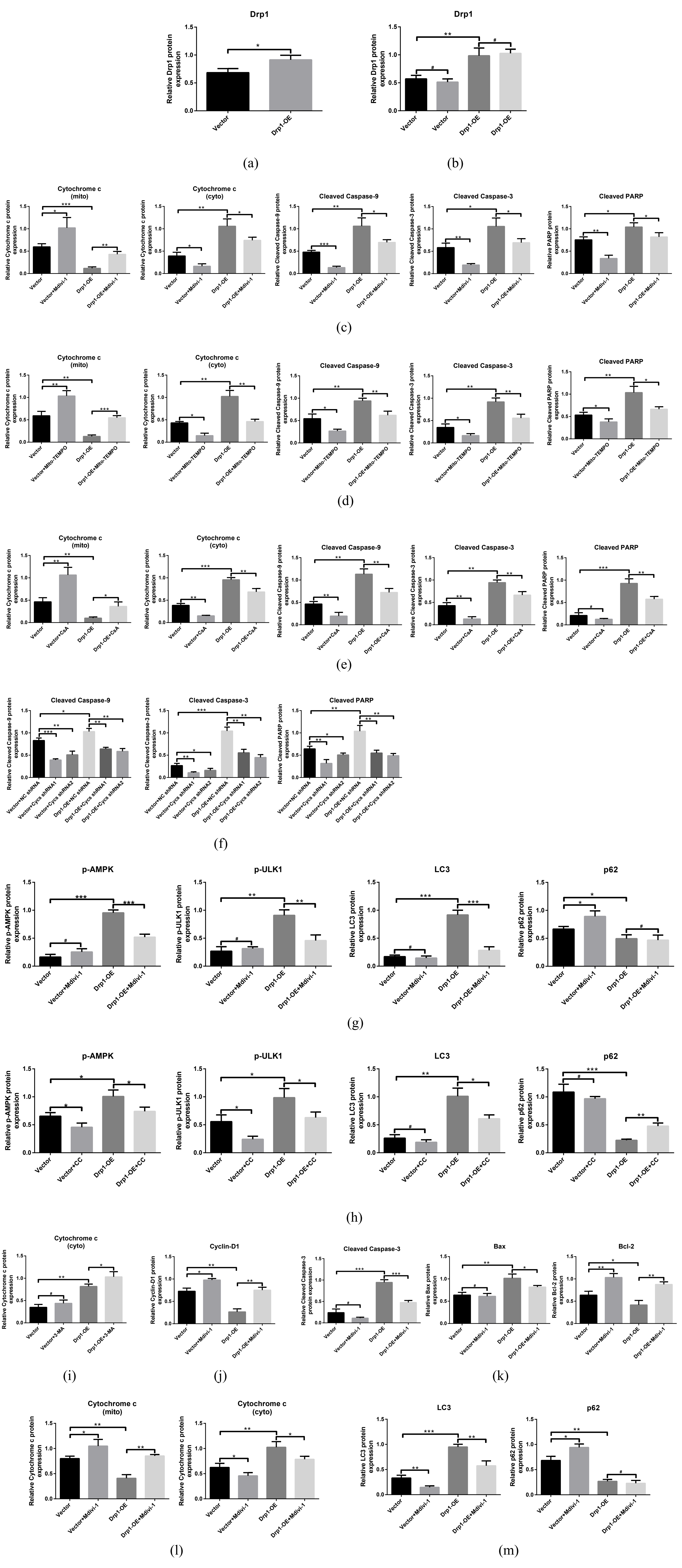
Drp1
Relative Drp1 protein expression
Vector
Drp1-OE
(a)
Drp1
Relative Drp1 protein expression
Vector
Drp1-OE
(b)
Cytochrome c (mito)
Cytochrome c (cyto)
Cleaved Caspase-9
Cleaved Caspase-3
Cleaved PARP
Vector
Vector+Mdivi-1
Drp1-OE
Drp1-OE+Mdivi-1
(c)
Cytochrome c (mito)
Cytochrome c (cyto)
Cleaved Caspase-9
Cleaved Caspase-3
Cleaved PARP
Vector
Vector+Mito-TEMPO
Drp1-OE
Drp1-OE+Mito-TEMPO
(d)
Cytochrome c (mito)
Cytochrome c (cyto)
Cleaved Caspase-9
Cleaved Caspase-3
Cleaved PARP
Vector
Vector+CsA
Drp1-OE
Drp1-OE+CsA
(e)
Cleaved Caspase-9
Cleaved Caspase-3
Cleaved PARP
Vector+NC shRNA
Vector+Cycs shRNA1
Vector+Cycs shRNA2
Drp1-OE+NC shRNA
Drp1-OE+Cycs shRNA1
Drp1-OE+Cycs shRNA2
(f)
p-AMPK
p-ULK1
LC3
p62
Vector
Vector+Mdivi-1
Drp1-OE
Drp1-OE+Mdivi-1
(g)
p-AMPK
p-ULK1
LC3
p62
Vector
Vector+CC
Drp1-OE
Drp1-OE+CC
(h)
Cytochrome c (cyto)
Vector
Vector+3-MA
Drp1-OE
Drp1-OE+3-MA
(i)
Cyclin-D1
(j)
Cleaved Caspase-3
Bax
Bcl-2
(k)
Cytochrome c (mito)
Cytochrome c (cyto)
(l)
LC3
p62
(m)

Figure S3

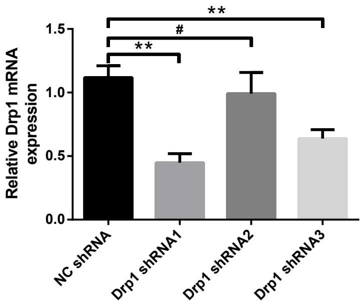

(a)

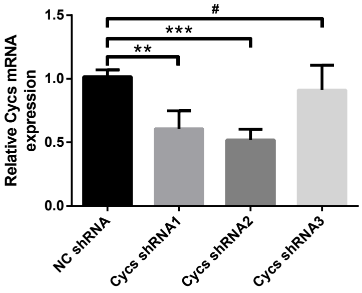

(b)

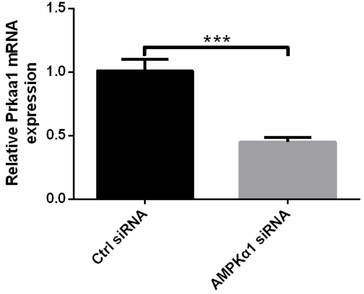

(c)

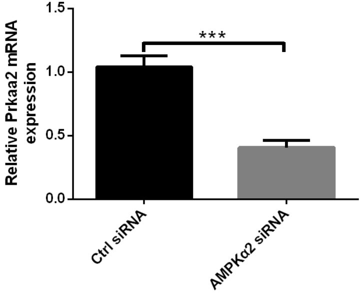

(d)

Figure S4

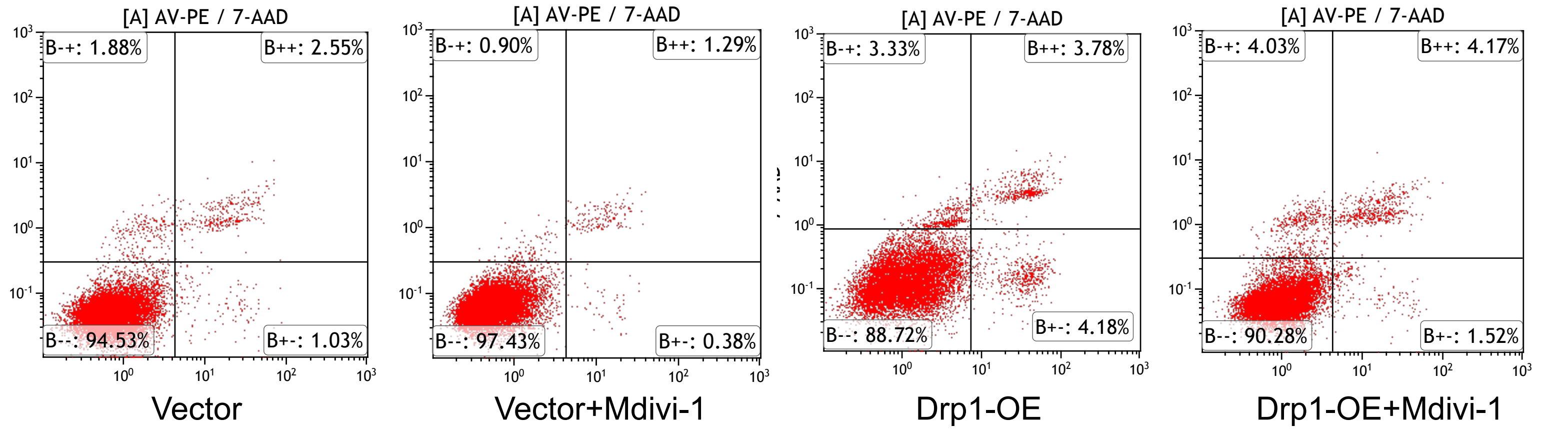

(a)

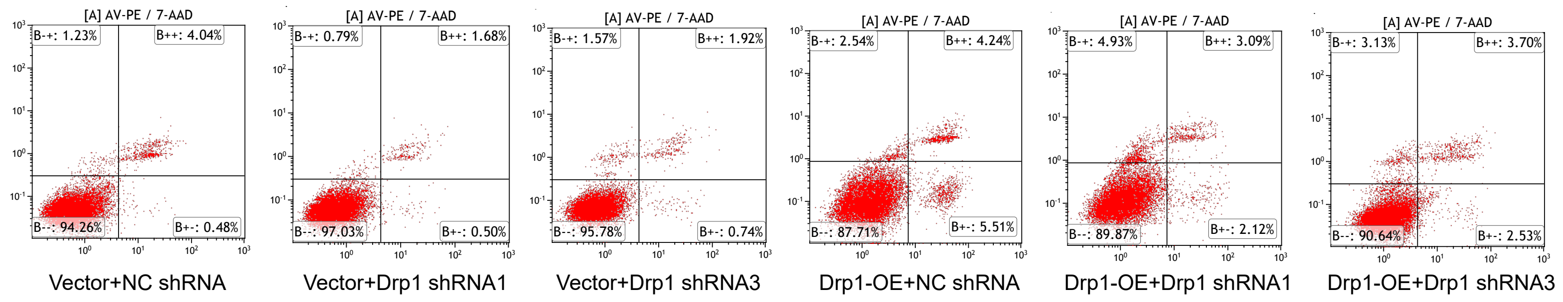

(b)

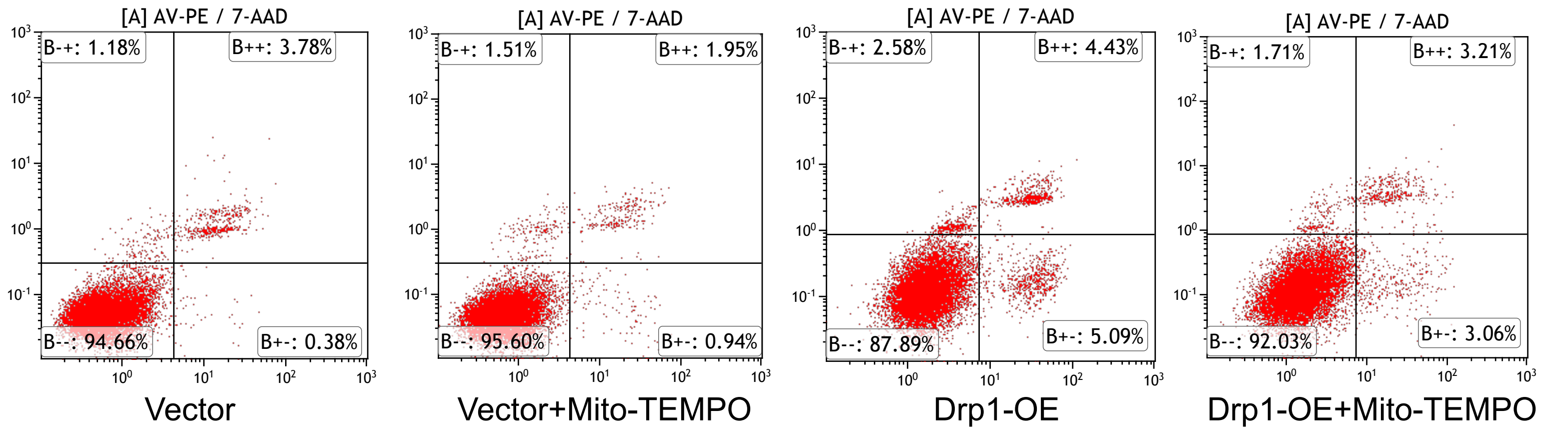

(c)

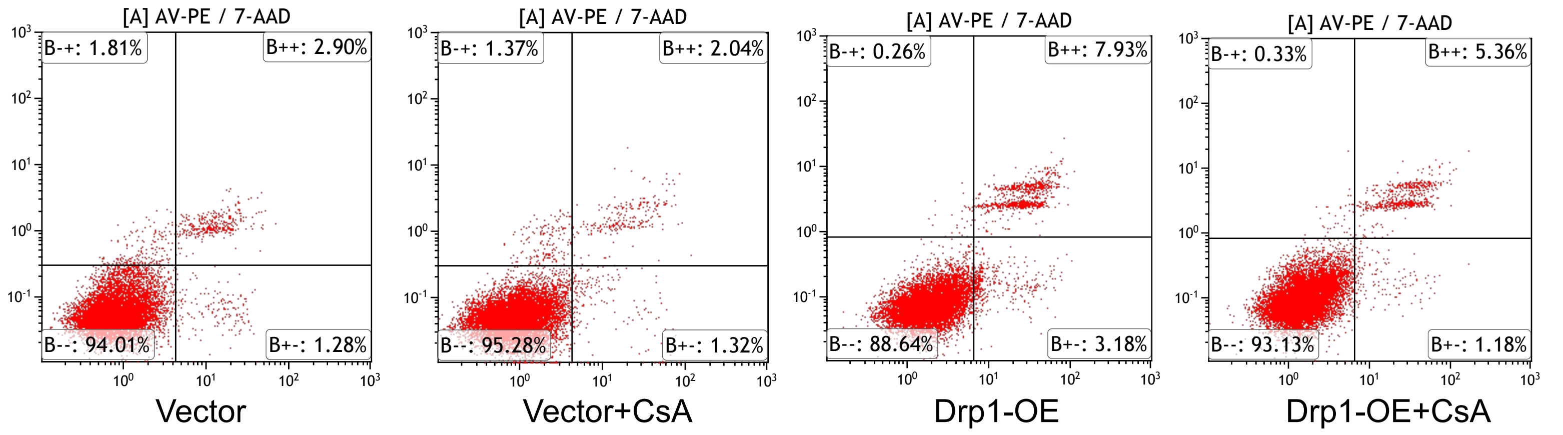

(d)

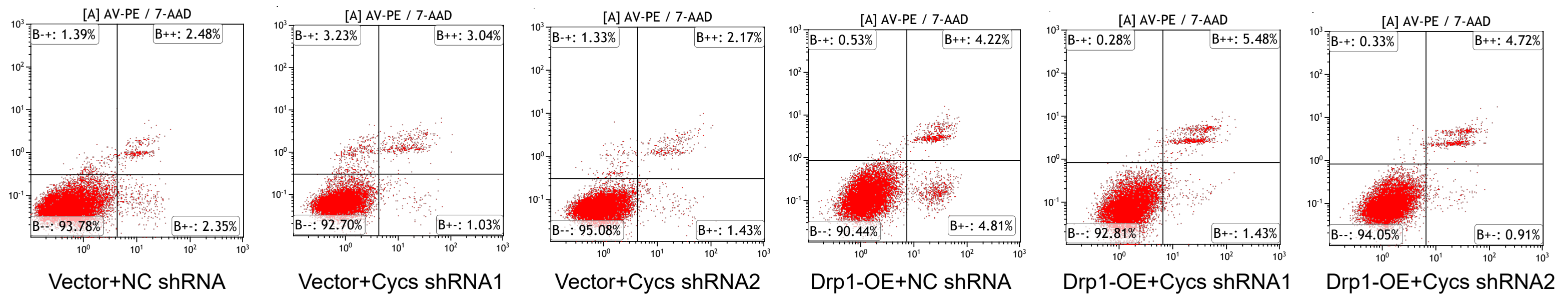

(e)

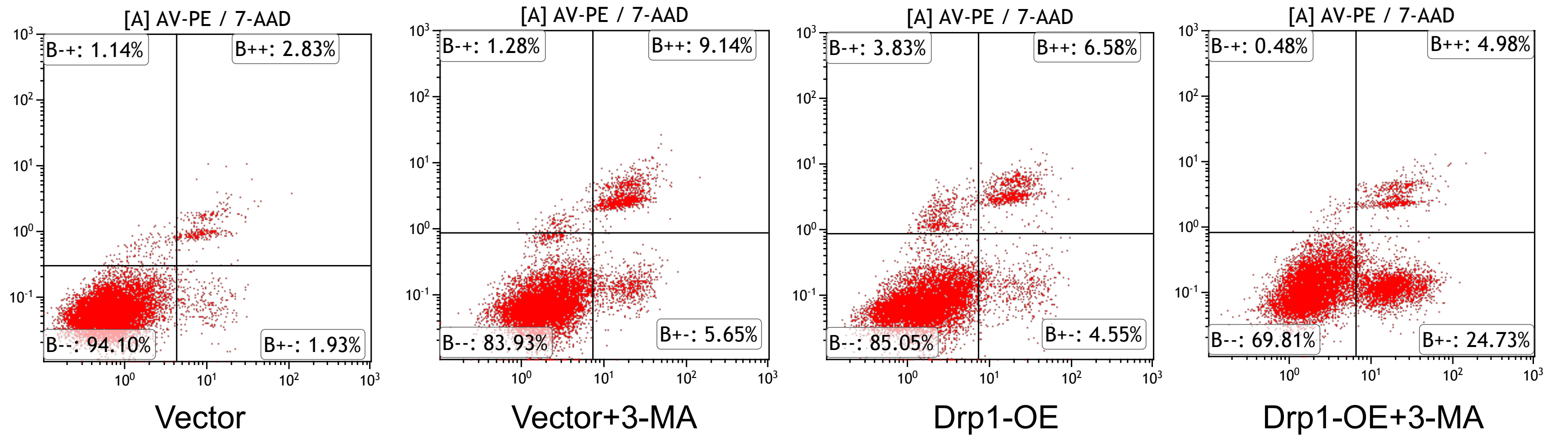

(f)

Figure S5

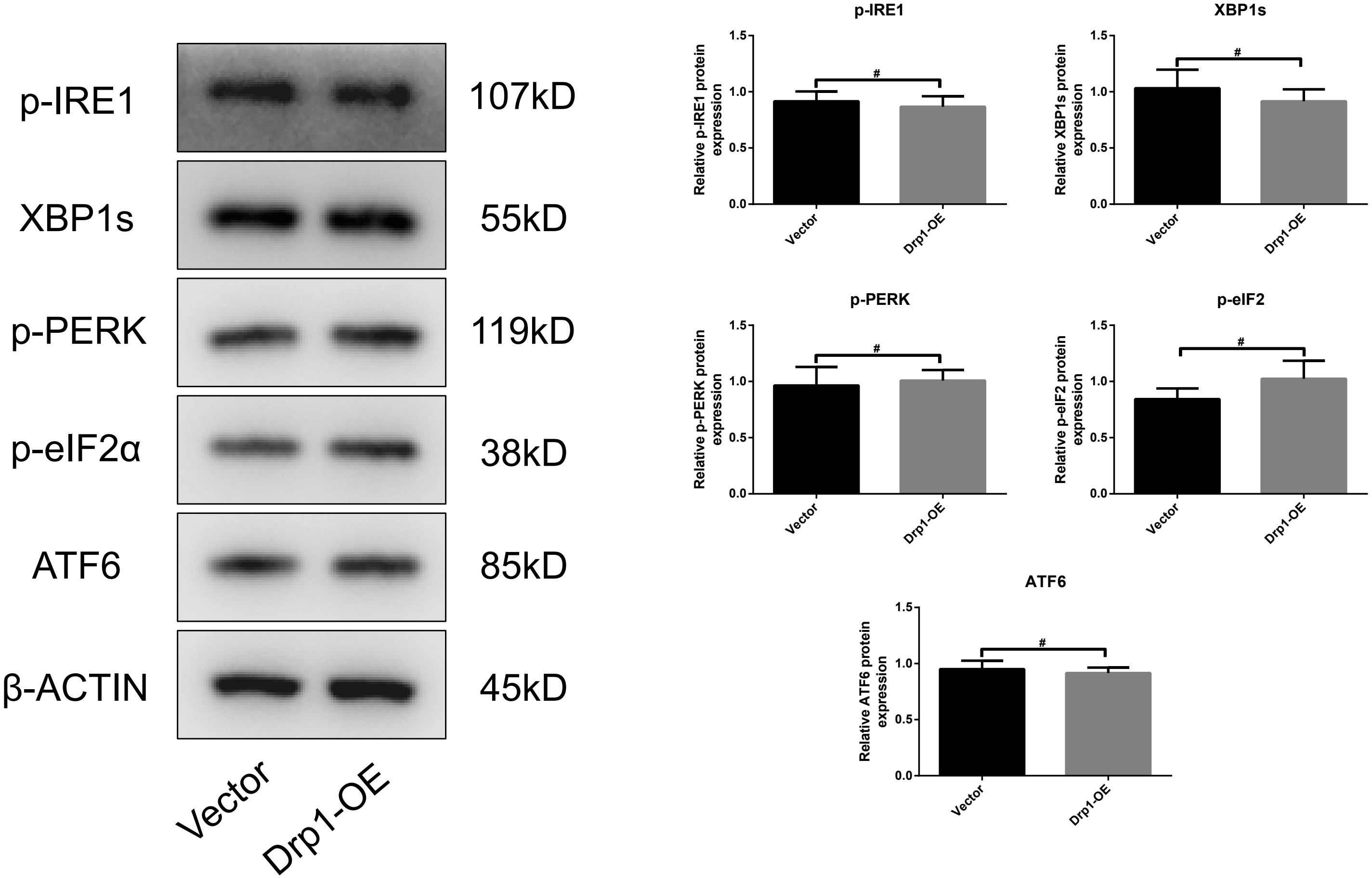

p-IRE1
XBP1s
p-PERK
p-eIF2α
ATF6
β-ACTIN
107kD
55kD
119kD
38kD
85kD
45kD
Vector
Drp1-OE
p-IRE1
XBP1s
p-PERK
p-eIF2
ATF6
Relative p-IRE1 protein expression
Relative XBP1s protein expression
Relative p-PERK protein expression
Relative p-eIF2 protein expression
Relative ATF6 protein expression
0.0
0.5
1.0
1.5
#

Figure S6

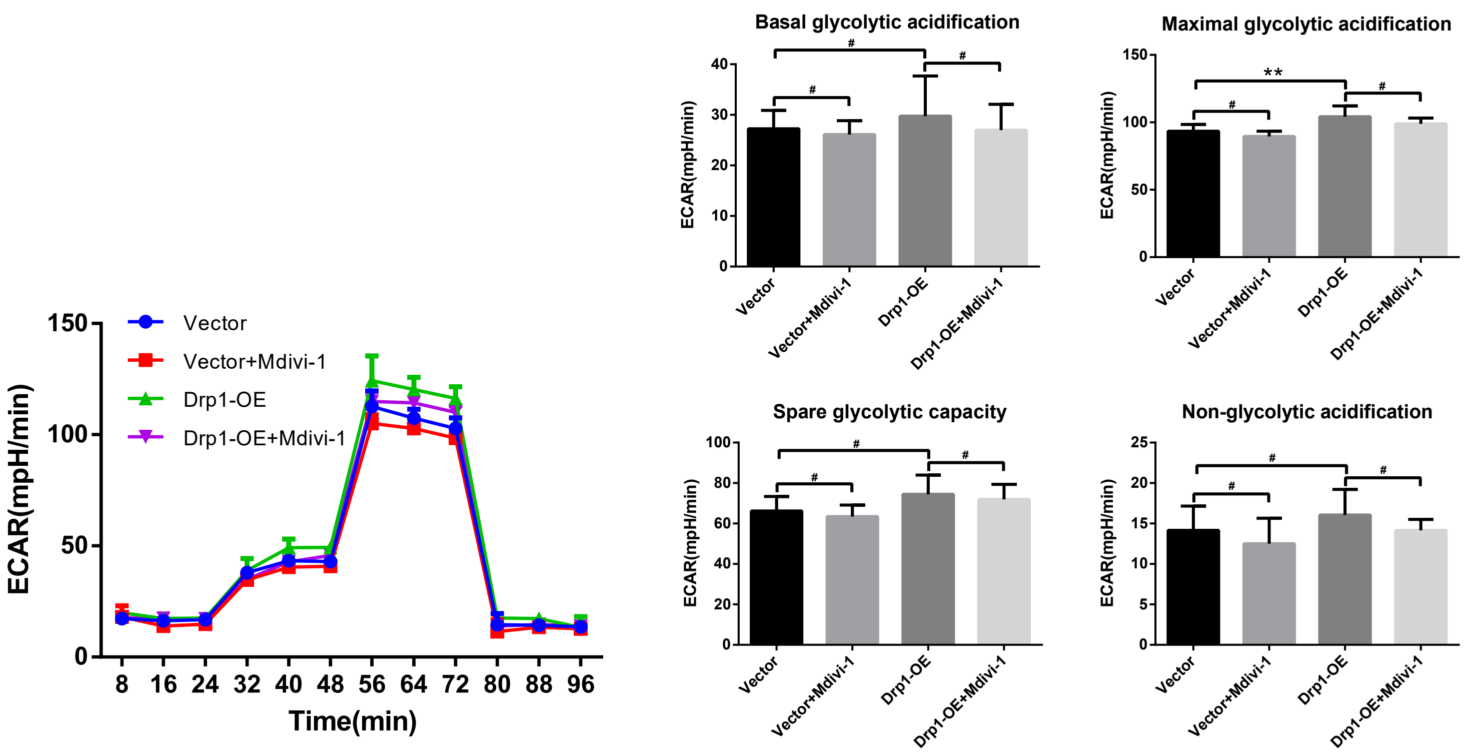

Vector
Vector+Mdivi-1
Drp1-OE
Drp1-OE+Mdivi-1
ECAR(mpH/min)
Time(min)
Basal glycolytic acidification
Maximal glycolytic acidification
Spare glycolytic capacity
Non-glycolytic acidification
#
**

(a)

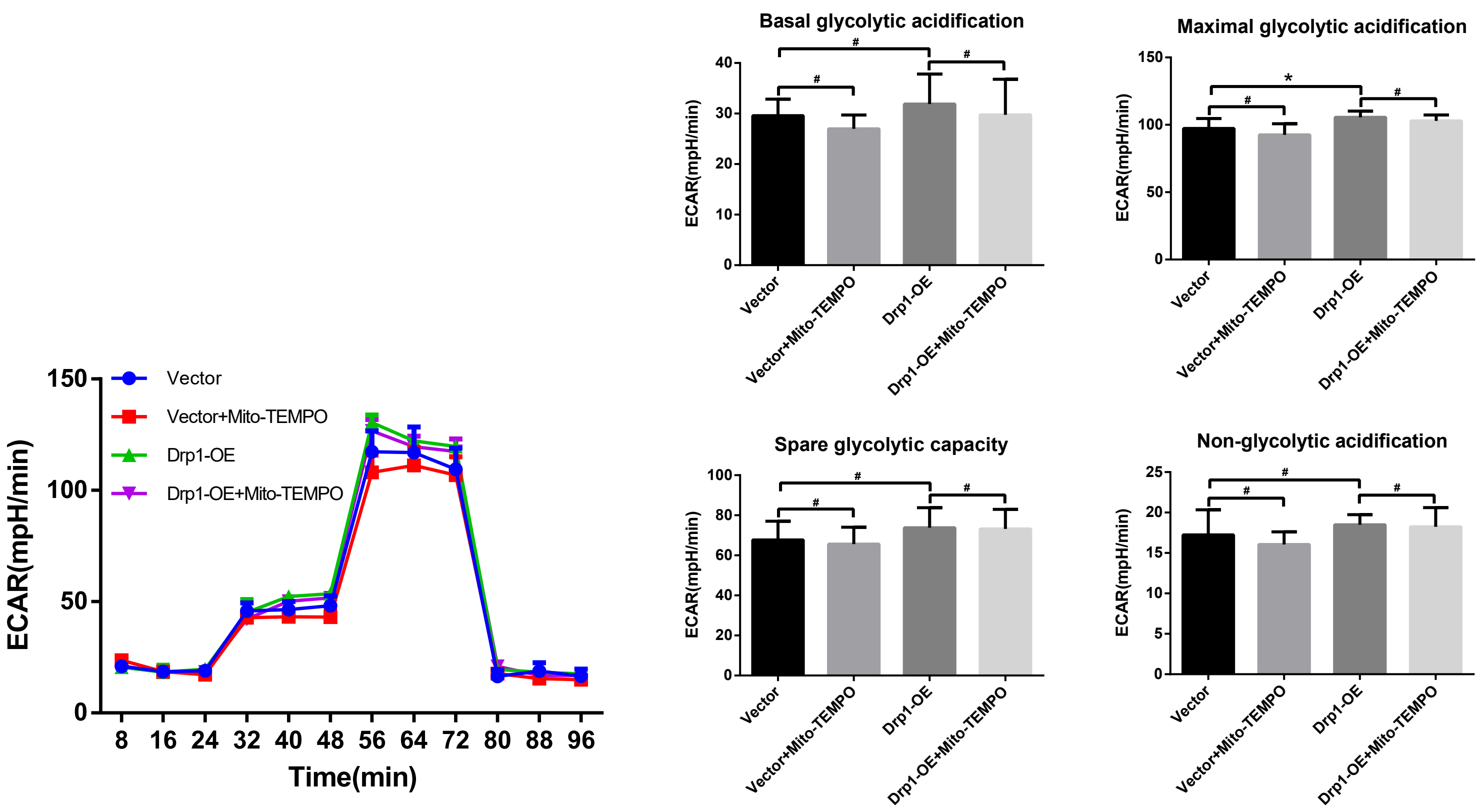

Vector
Vector+Mito-TEMPO
Drp1-OE
Drp1-OE+Mito-TEMPO
ECAR(mpH/min)
Time(min)
Basal glycolytic acidification
Maximal glycolytic acidification
Spare glycolytic capacity
Non-glycolytic acidification
#
*

(b)

Figure S7

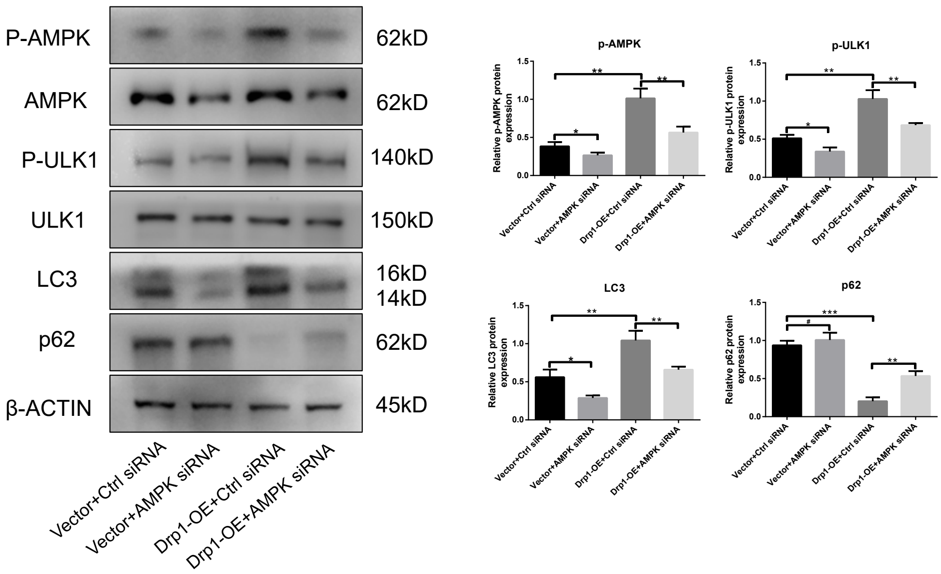

P-AMPK
AMPK
P-ULK1
ULK1
LC3
p62
β-ACTIN
62kD
62kD
140kD
150kD
16kD
14kD
62kD
45kD
Vector+Ctrl siRNA
Vector+AMPK siRNA
Drp1-OE+Ctrl siRNA
Drp1-OE+AMPK siRNA
p-AMPK
Relative p-AMPK protein expression
p-ULK1
Relative p-ULK1 protein expression
LC3
Relative LC3 protein expression
p62
Relative p62 protein expression

(a)

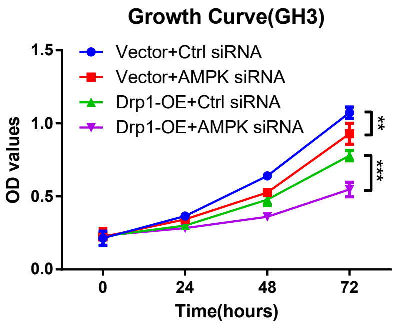

Growth Curve(GH3)
Vector+Ctrl siRNA
Vector+AMPK siRNA
Drp1-OE+Ctrl siRNA
Drp1-OE+AMPK siRNA
OD values
Time(hours)
0
24
48
72
1.5
1.0
0.5
0.0
**
***

(b)

Figure S8

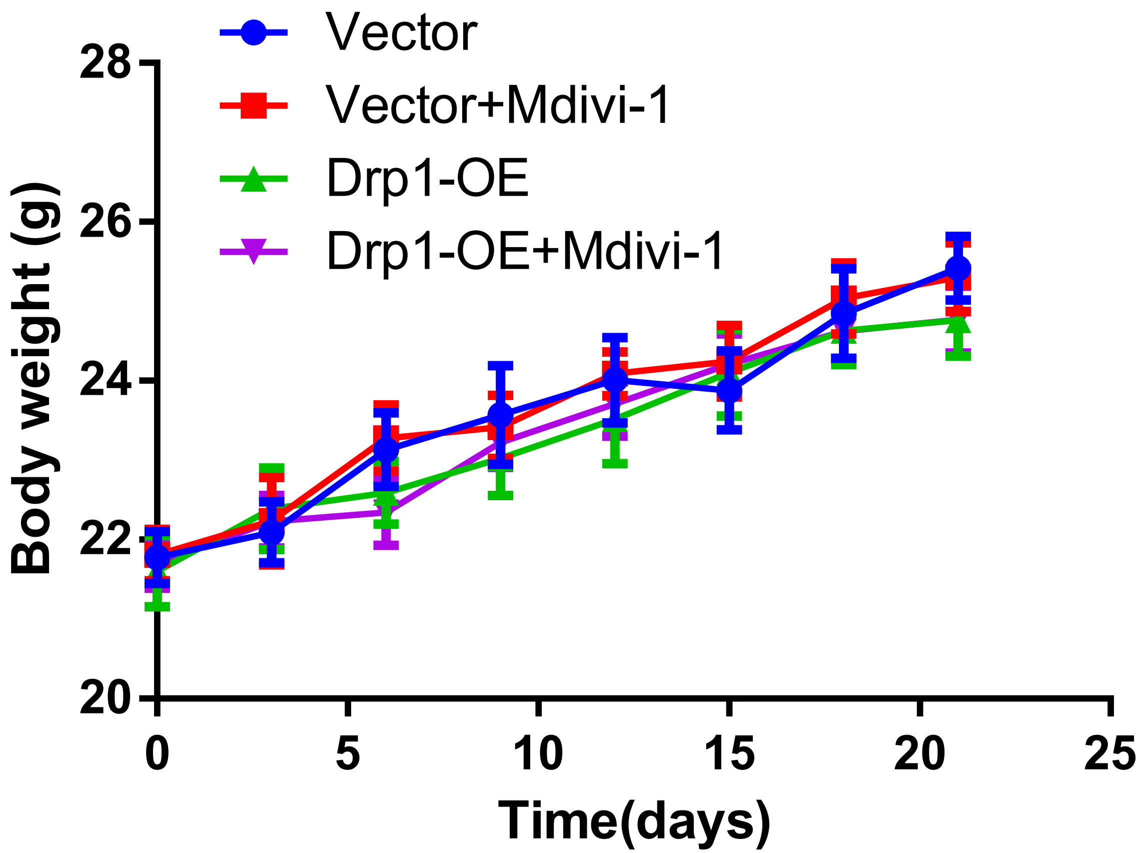

Vector
Vector+Mdivi-1
Drp1-OE
Drp1-OE+Mdivi-1
Body weight (g)
28
26
24
22
20
0
5
10
15
20
25
Time(days)
